# Socioeconomic inequalities in outcomes, experiences and treatment among adults consulting primary care for a musculoskeletal pain condition: a prospective cohort study

**DOI:** 10.1136/bmjopen-2024-095132

**Published:** 2025-07-15

**Authors:** George M Peat, Jonathan C Hill, Dahai Yu, Simon Wathall, Emma Parry, James Bailey, Clare Thompson, Kelvin P Jordan, The MIDAS Patient Advisory Group, Lindsey Brown

**Affiliations:** 1 Centre for Applied Health & Social Care (CARe), Sheffield Hallam University, Sheffield, UK; 2 Primary Care Centre Versus Arthritis, School of Medicine, Keele University, Keele, UK

**Keywords:** Musculoskeletal disorders, Chronic Pain, Primary Health Care, Health Equity, Patient Reported Outcome Measures, Health Services

## Abstract

**Abstract:**

**Objectives:**

To estimate the direction and magnitude of socioeconomic inequalities in outcome, experience and care among adults consulting for a musculoskeletal pain condition.

**Design:**

Multicentre, prospective observational cohort with repeated measures at three waves (baseline, 3 months and 6 months after index consultation).

**Setting:**

30 general practices in North Staffordshire and Stoke-on-Trent, England.

**Participants:**

1875 consecutive, eligible, consenting patients, aged 18 years and over, presenting with a relevant SNOMED CT-coded musculoskeletal pain condition between September 2021 and July 2022.

**Interventions:**

Standard care.

**Primary and secondary outcome measures:**

Primary outcome was patient-reported pain and function using the Musculoskeletal Health Questionnaire (MSK-HQ score, 0–56). Secondary outcomes were patient experience (overall dissatisfaction with consultation experience, dichotomised) and an indicator of care received (opioid prescription within 14 days of index consultation). Using multilevel models, we examined inequalities in primary and secondary outcomes by area deprivation (Index of Multiple Deprivation derived from patient residential postcode), before and after adjusting for sociodemographic and survey administration variables, clinical case-mix and selected practice-level covariates.

**Results:**

Compared with patients from the least deprived neighbourhoods, patients from the most deprived neighbourhoods had significantly poorer MSK-HQ scores at baseline (mean 22.6 (SD 10.4) vs 27.6 (10.1)). At 6 months, the inequality gap in MSK-HQ score widened (difference in mean score after adjustment for all covariates: 1.94; 95% CI: −0.70 to 4.58). Opioid prescription was more common for patients living in the most deprived neighbourhoods (30% vs 19%; fully adjusted OR: 0.69; 95% CI: 0.44 to 1.08). Only 6% of patients overall reported being dissatisfied with their consultation. Analysis of multiply imputed data produced a similar pattern of findings to complete-case analysis.

**Conclusions:**

Substantial inequalities in the chronicity, severity and complexity of musculoskeletal pain problems are already present at the time of accessing care. Inequalities in pain and function do not reduce after accessing care and may even widen slightly.

**Trial registration number:**

ISRCTN18132064; Results.

**STRENGTHS AND LIMITATIONS OF THIS STUDY**

- Prospectively designed and registered study of ‘real world outcomes’ featured the collection and linkage of key outcomes and multilevel determinants from multiple data sources across diverse practices within a single integrated care system.
- Inequalities in patient outcomes estimated before and after clinical case-mix adjustment.
- A patient advisory group was involved throughout the design, conduct and interpretation of the study.
- Selective non-participation, timing of ‘baseline’ measurement and loss to follow-up could potentially bias our findings. Practitioner-level variation and determinants were not included.

## Introduction

 Musculoskeletal pain conditions—low back pain, neck pain, osteoarthritis and other regional pain conditions—are a major cause of population disability,^1^ typically occur more frequently and have a greater impact in socioeconomically deprived individuals and communities.^2^ Evidence from previous studies in the UK suggests that healthcare may follow a Disproportionate Care Law^3^ in which people from more deprived backgrounds may have higher healthcare use but the care is of lower quality and insufficient for their additional complexity and less favourable prognosis at the time of presenting to services. For example, compared with the least deprived areas, people living in the most deprived neighbourhoods in England have consistently higher primary care consultation rates for musculoskeletal pain conditions.^4^ They also present with higher levels of severity, complexity and comorbidity,^5^ and they are more likely to be treated with long-term opioid analgesia.^6^,^9^

The UK Health and Social Care Act 2022 introduced a legal duty on the National Health Service (NHS) to consider how to reduce health inequalities.^10^ This legal duty is not just in relation to access to services but also to the outcomes achieved by the provision of health services, specifically, inequalities in effectiveness, safety and patient experience. However, a lack of appropriate data within the same well-defined population presents an obstacle to understanding the nature and extent of any outcome inequalities. Data routinely collected in primary care cover healthcare contacts, prescriptions and other processes of care, but seldom the patient-reported measures of severity, outcomes, experience and case-mix adjustment characteristics required to move beyond simple descriptions of patterns of care.

In this prospective cohort study of adults presenting to primary care with a musculoskeletal pain condition, we designed, collected and analysed linked data from a local data integration pilot project to examine whether outcome inequalities reduce or widen after accessing primary care, and whether inequalities in patient experience and receipt of low value care are also seen. We focused on general practice (GP) as the healthcare setting, where the majority of assessment and management of these conditions takes place, and on inequalities by area-level deprivation. Health and care for adults living in the most deprived 20% of neighbourhoods is a key focus in the current NHS England CORE20PLUS5 strategy for reducing healthcare inequalities.^11^ Our specific objective was to estimate the magnitude and direction of differences between groups of patients defined by deprivation in the following outcomes: (1) patient-reported musculoskeletal health status (including pain and function) up to 6 months following index consultation, (2) overall level of satisfaction with experience of consulting primary care for their musculoskeletal problem and (3) receipt of an opioid analgesic. We further explored whether any observed outcome inequalities were still evident after accounting for differences in demographic characteristics, clinical case-mix adjustment characteristics and in staffing levels of GPs and non-medical healthcare professionals involved in direct patient care (DPC) in the GP.

## Methods

This was a prospective cohort study of musculoskeletal consulters with survey waves at baseline (responding approximately 2 weeks after consultation), 3 months and 6 months and linkage to primary care electronic health records (EHRs). The study was registered prospectively (ISRCTN18132064), the pre-specified protocol was published in the Open Science Framework repository (https://osf.io/e542w/#!) and is reproduced in online supplemental appendix 1. Ethical approval was received from Yorkshire and The Humber – Leeds West Research Ethics Committee (RG-0327–21). The report was prepared with reference to the Strengthening the Reporting of Observational Studies in Epidemiology^12^ and Guidance for Reporting Involvement of Patients and the Public^13^ reporting guidelines (onlinesupplemental appendices 6  7).

### Setting

The MIDAS-GP study was set in GPs located in North Staffordshire and Stoke-on-Trent, UK, within the Staffordshire and Stoke-on-Trent Integrated Care System. At the time of the study, there were 70 GPs within 13 primary care networks (PCNs) in this area. Eligible practices had a computer system and text-messaging service suitable for patient identification and recruitment. We aimed for a minimum of 26 GPs covering all PCNs and attempted to over-sample practices located in more socioeconomically deprived communities or serving the most ethnically diverse populations.

### Study population

The eligible study population was patients aged 18 years and over with a recorded SNOMED CT code for a musculoskeletal painful condition during each practice’s recruitment period. A list of SNOMED CT Concept IDs suitable for practical application in UK primary care data was developed for this purpose (the full process is detailed at https://osf.io/e542w/#!). The final inclusion list contained 498 Concept IDs. Patients with an inflammatory condition code recorded within the previous 3 years were excluded. Code lists for musculoskeletal pain and inflammatory conditions are in onlinesupplemental appendices 2  3.

Staff from the NIHR Clinical Research Network: West Midlands worked with practice staff to perform weekly/fortnightly searches of the practice EHR system. Recruitment took place between September 2021 and July 2022, staggered across practices. Eligible patients with an active mobile telephone number were sent a short messaging service text from the practice computer system and asked to complete a web-based questionnaire. Patients without an active mobile telephone number at the practice were posted a questionnaire for pen-and-paper completion. Respondents were asked for informed consent (electronic or written) to link their self-report survey responses to their primary care EHRs and to be sent follow-up questionnaires at 3 months and 6 months.

### Outcomes

The primary outcome was the patient-reported Musculoskeletal Health Questionnaire (MSK-HQ), a 14-item measure that captures key components of musculoskeletal health that have been prioritised by patients, including pain and function. Scores range from 0 to 56, with higher scores indicating better overall musculoskeletal health status over the past 2 weeks.^14^

Secondary outcomes were (1) overall dissatisfaction with their musculoskeletal consultation experience (dichotomised into fairly poor/very poor vs very good/good/neither good nor poor) reported in baseline (postconsultation) questionnaire and (2) prescription of an opioid analgesic within 14 days of their index musculoskeletal consultation recorded in the primary care EHR (code list for opioid analgesics in online supplemental appendix 4).

### Exposure

Patient deprivation was based on the English Index of Multiple Deprivation (IMD) 2019 for residential postcode.^15^ The IMD is a composite measure of neighbourhood deprivation covering seven domains (health deprivation and disability; barriers to housing and services; employment; income; education, skills and training; crime; living environment) and ranks each neighbourhood (lower super output area: mean population of 1500) from most to least deprived. Participants were categorised into five quintile groups from most to least deprived based on the national ranking of neighbourhoods.^16^

### Covariates

Covariates were selected based on a directed acyclic graph constructed a priori.

#### Sociodemographic and survey-related covariates

Age, sex, ethnicity, time between index consultation and baseline survey completion (days) and mode of survey administration (online or paper-based).

#### ‘Clinical case-mix’ covariates

Previous musculoskeletal surgery, duration of symptoms (<3 months, 4–6 months, 7–12 months, 13 months–3 years, over 3 years), number of pain sites, previous episodes of pain (0, 1, 2–3, 4–9, 10+), days of moderate physical activity in previous week (0–7), body mass index (BMI: kg/m^2^) and count of selected comorbidities (0, 1, 2, 3+). The count of comorbidities was derived from the primary care EHR in the 5 years prior to index MSK consultation. The list of comorbidities used was produced after cross-mapping morbidities in National Institute for Health and Care Excellence multimorbidity indicator for GP,^17^ Charlson^18^ and Elixhauser^19^ comorbidity indices and potentially relevant case-mix adjustment methods.^20 21^ Comorbidity code lists are available at Open Science Framework (https://osf.io/e542w/#!).

#### Practice-level covariates

Number of GP full-time equivalents (FTE) per 1000 registered population, total DPC staff FTE per 1000 registered population and musculoskeletal consultation rate.

### Sample size

We based our sample size on the following assumptions: the median registered population size for general practices in North Staffordshire and Stoke-on-Trent is 7300; 80% of registered patients are aged 18 years and over; the annual MSK consultation prevalence is 15%,^22^ a 25% participation rate; an 80% consent rate to EHR linkage; 50% follow-up at 6 months; each of 26 GP practices would recruit for a 3-month period. Based on these estimates and assumptions, we anticipated 1424 participants at baseline, 1139 consenting to EHR linkage and 569 responding at 6-month follow-up. This would allow detection of a difference on the follow-up MSK-HQ of 3 or more points (assuming SD of 10 points),^23^ with 80% power at the 5% significance level, for groups defined by a dichotomous covariate, two follow-up time points (3 months and 6 months), adjustment for baseline MSK-HQ score and assumed correlations of 0.5 between the two follow-up scores and between the follow-up and baseline scores. The length of the recruitment period was allowed to vary between practices in an attempt to reach a minimum of 50 baseline participants per practice and 100–150 per PCN.

### Analysis

Descriptive characteristics were compared for all those responding at baseline, those who both responded at baseline and agreed to EHR linkage and those responding to all three waves and agreed to EHR linkage.

For the primary outcome of MSK-HQ score, 3-level (wave, participant, GP) random intercept multilevel linear regression models were used. Absolute difference in MSK-HQ score between the five deprivation groups was determined: (a) unadjusted, (b) adjusted for sociodemographic and survey covariates, (c) additionally adjusted for clinical case-mix covariates and (d) further adjustment for the practice-level covariates. Age, time between consultation and baseline survey, BMI, days of physical activity and the practice-level covariates were mean-centred. Each model included a ‘wave × deprivation’ interaction term to explore whether any inequality gaps in MSK-HQ changed across time. The extent of unexplained variation at each level was determined by first fitting a variance component model (no explanatory variables included) and deriving the intracluster correlation coefficient and then repeating for each model (a)–(d).

Analyses were repeated using two-level (participant, GP) multilevel logistic regression models for the secondary outcomes of dissatisfaction with consultation and opioid prescription.

The primary analyses used the observed data from baseline responders with linkage to EHR. We described baseline characteristics among complete cases and total eligible respondents to judge whether the complete case dataset could reasonably be judged as representative of the total eligible respondent dataset (ie, data missing completely at random). In sensitivity analysis, we assumed data were missing at random and used multiple imputation to impute information for those not responding at one or both follow-ups and for the EHR covariates for responders without linkage. Considering a scenario where 50% of participants had one or more missing data points for exposure, covariates or outcomes, we generated 50 imputed datasets. Throughout the imputation process, we preserved the longitudinal nature of the data. Multilevel multiple imputation was employed to create these datasets. We developed separate imputation models for each of the three outcomes—MSK-HQ, dissatisfaction with consultation and opioid prescription—due to their differing longitudinal structures (3-level for MSK-HQ and 2-level for dissatisfaction and opioid prescription). The final estimates were derived using the chained equations approach, following Rubin’s rules.

### Patient and public involvement

Two members of the public were coapplicants in the funding application for the MIDAS research programme. On successful award, this was expanded to a dedicated public advisory group (PAG) comprising seven people with lived experience of musculoskeletal conditions drawn from Keele University’s Research User Group. The PAG met with the MIDAS programme lead, chief investigators, trial manager and other members of the research team on a monthly basis via Microsoft Teams from the very start of the award. PAG members advised on the design of the study, specifically reviewing, discussing and proposing revisions to: (1) planned recruitment procedures to raise awareness and inform potential participants of the purpose of the study, (2) modes of survey administration to widen accessibility of the study and reduce selection bias, for example, offering pen-and-paper completion, highlighting the ability to access language translation services. and get help from family members, (3) questionnaire content, length and order and (4) consent procedures. Initial findings were shared with PAG members who contributed to the interpretation of the findings. PAG members are involved in planning dissemination aimed at patient and public audiences. The MIDAS programme also had an independent advisory board with public representation that met biannually to offer critical advice on research plans and findings.

## Results

**Figure 1:**
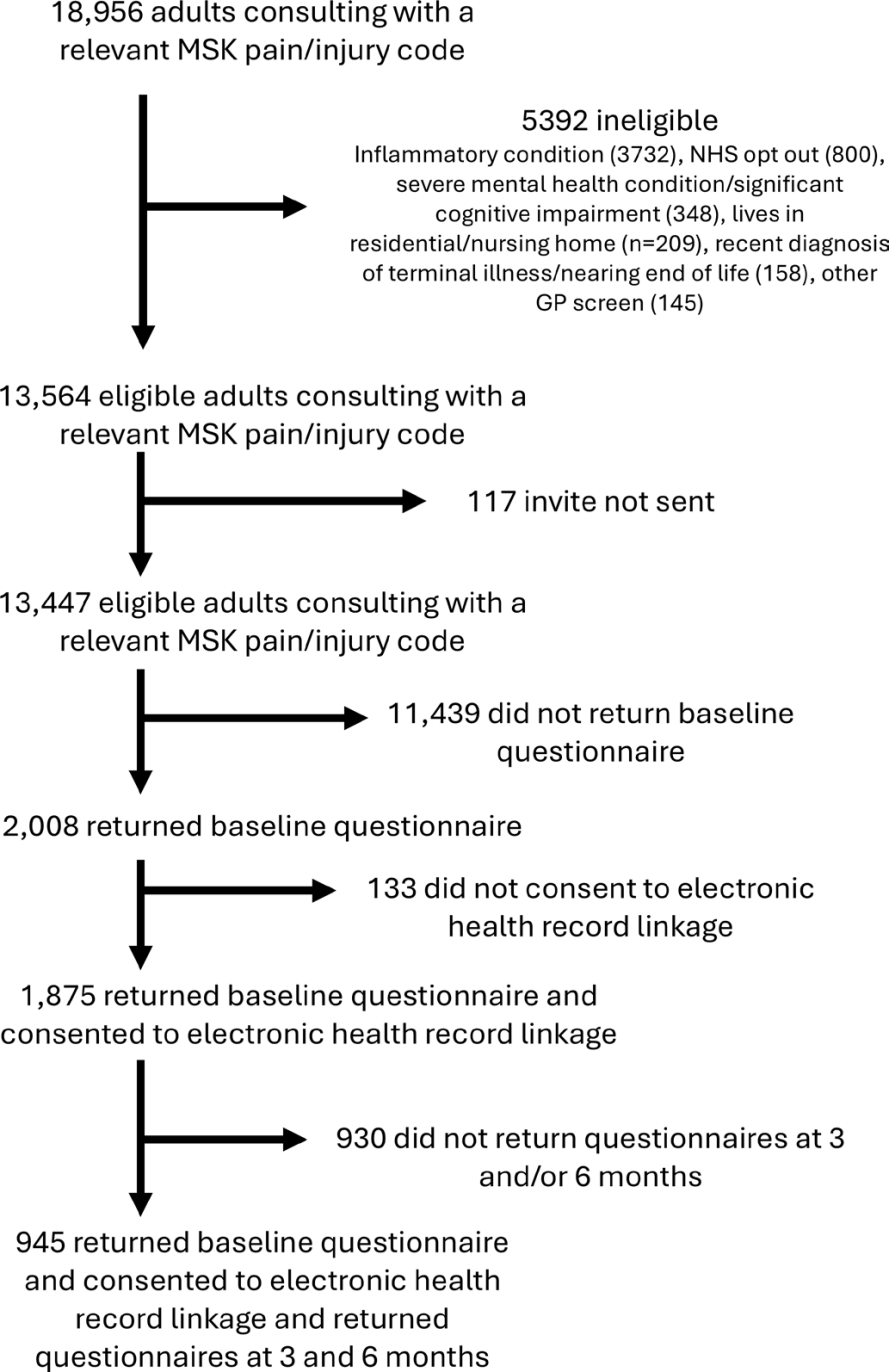
Participant flowchart. GP, general practice; MSK, musculoskeletal; NHS, National Health Service.

13 447 patients were invited to participate from 30 GPs, ensuring at least one practice participated from each of the 13 PCNs and enabling less research-experienced practices serving more diverse populations to take part. 2008 (14.9%) patients responded at baseline, of which 1875 consented and were successfully linked to their EHR and hence form the primary population for analysis. Of these 1875, 945 (50%) responded to all three data time points (waves) (figure 1). The mean age was 57.7 years (SD 15.5), 1223 (66%) of them were women, 530 (28%) lived in the most deprived quintile (20%) of neighbourhoods nationally and 1788 (95%) self-reported their ethnicity as white (table 1). Participants from the most deprived neighbourhoods had a younger mean age and a higher proportion self-reported their ethnic background as black, Asian, Mixed/multiple or ‘other’ and were also more likely to have longstanding pain, more previous pain episodes, multiple site pain and comorbidities.

**Table 1:** Characteristics of participants, overall and by deprivation status

	Total	Area-level deprivation*				
		IMDq1 (most)	IMDq2	IMDq3	IMDq4	IMDq5 (least)
N	1875	530	383	398	320	244
Age, mean (SD)	57.7 (15.5)	51.4 (15.2)	57.3 (14.6)	61.2 (15.1)	61.7 (14.7)	61.2 (14.8)
Female	1233 (66)	350 (66)	260 (68)	267 (67)	194 (61)	162 (66)
BMI, mean (SD)	29.2 (6.9)	30.1 (8.2)	29.8 (7.1)	29.0 (6.1)	28.6 (6.0)	27.3 (5.4)
Ethnicity						
Asian	31 (2)	18 (3)	6 (2)	5 (1)	1 (<1)	1 (<1)
Black	28 (2)	20 (4)	5 (1)	2 (1)	1 (<1)	0 (0)
Mixed/multiple	11 (1)	3 (1)	4 (<1)	3 (1)	0 (0)	1 (<1)
Other	17 (1)	6 (1)	6 (2)	1 (<1)	1 (<1)	3 (1)
White	1788 (95)	483 (91)	362 (95)	387 (97)	317 (99)	238 (98)
Time between consultation and survey (days), mean (SD)	9.4 (11.1)	9.2 (18.9)	9.6 (15.1)	9.8 (8.4)	10.8 (14.5)	8.9 (7.0)
Paper survey	282 (15)	55 (10)	54 (14)	72 (18)	56 (18)	45 (18)
Previous MSK surgery	238 (13)	66 (12)	49 (13)	46 (13)	46 (14)	31 (13)
Duration of symptoms						
<3 months	758 (40)	219 (41)	150 (39)	171 (43)	122 (38)	90 (37)
4–6 months	205 (11)	50 (9)	40 (10)	48 (12)	38 (12)	29 (12)
7–12 months	251 (13)	64 (12)	54 (14)	55 (14)	40 (16)	38 (16)
13 months–3 years	290 (15)	75 (14)	67 (17)	55 (14)	53 (17)	40 (16)
>3 years	371 (20)	122 (23)	70 (18)	67 (17)	67 (21)	45 (18)
Previous episodes						
0	345 (18)	73 (14)	64 (17)	83 (21)	55 (17)	53 (22)
1	129 (7)	27 (5)	21 (6)	36 (9)	28 (9)	17 (7)
2–3	306 (16)	80 (15)	69 (18)	67 (17)	51 (16)	39 (16)
4–9	233 (12)	70 (13)	47 (12)	54 (14)	36 (11)	26 (11)
10+	862 (46)	276 (52)	179 (47)	154 (39)	147 (46)	106 (43)
Pain sites						
1 site	936 (50)	243 (46)	190 (50)	217 (54)	155 (48)	128 (53)
2 sites	390 (21)	92 (17)	91 (24)	71 (18)	72 (23)	64 (26)
≥3 sites	549 (29)	195 (37)	101 (26)	110 (28)	92 (29)	51 (21)
Physical activity (days), mean (SD)	2.2 (2.4)	2.1 (2.5)	2.1 (2.3)	2.2 (2.4)	2.1 (2.5)	2.3 (2.5)
Comorbidity count						
0	829 (44)	224 (42)	163 (43)	186 (47)	134 (42)	122 (50)
1	577 (31)	150 (28)	121 (32)	123 (31)	106 (33)	77 (32)
2	329 (18)	108 (20)	64 (17)	60 (15)	60 (19)	37 (15)
≥3	140 (7)	48 (9)	35 (9)	29 (7)	20 (6)	8 (3)
DPC FTE per 1000 registered population, mean (SD)	21.8 (13.7)	20.7 (13.3)	21.4 (13.1)	23.1 (12.9)	24.3 (14.2)	19.4 (15.5)
GP FTE per 1000 registered population, mean (SD)	60.4 (27.5)	54.1 (25.8)	67.5 (29.6)	61.0 (27.6)	63.2 (24.2)	57.9 (28.5)
Practice-level MSK consultation per 10 000, mean (SD)	2683 (845)	2532 (44)	2598 (787)	2712 (830)	2747 (738)	3017 (1144)
Responded at 3 months	1323 (71)	350 (66)	261 (68)	283 (71)	241 (75)	188 (77)
Responded at 6 months	1280 (68)	314 (59)	266 (69)	283 (71)	240 (75)	177 (73)
Baseline MSK-HQ, mean (SD)	25.8 (10.6)	22.8 (10.4)	26.1 (10.7)	26.5 (10.5)	27.2 (10.7)	27.6 (10.1)
Month-3 MSK-HQ, mean (SD)	30.2 (12.7)	26.9 (13.1)	29.2 (12.5)	31.6 (12.7)	31.5 (11.9)	32.9 (12.4)
Month-6 MSK-HQ, mean (SD)	31.4 (13.3)	27.0 (12.8)	30.9 (13.4)	33.2 (13.8)	32.8 (12.5)	34.3 (12.9)
MSK-HQ MIC at 3 months	375 (40)	87 (38)	60 (32)	93 (44)	78 (42)	57 (42)
MSK-HQ MIC at 6 months	415 (44)	88 (39)	74 (40)	108 (51)	76 (41)	69 (51)
Dissatisfaction with consultation	119 (6)	47 (9)	23 (6)	22 (6)	13 (4)	14 (6)
Opioid analgesic	493 (26)	158 (30)	99 (26)	114 (29)	75 (23)	47 (19)

Numbers are n (%) unless otherwise stated.

Patients meeting or exceeding the minimal important change value for the MSK-HQ (5.5 points(38)) compared with baseline.

* Index of Multiple Deprivation (IMD) available for n=1961.

BMI, body mass index; DPC, direct patient care; FTE, full time equivalent; GP, general practitioner; IMDq1–q5, IMD grouped by quintile value, most deprived to least deprived; MIC, minimal important change; MSK, musculoskeletal; MSK-HQ, Musculoskeletal Health Questionnaire.

Overall, those responding at all three waves were slightly older and less likely to live in the most deprived neighbourhoods, but were similar on MSK-HQ at baseline, dissatisfaction with consultation and opioid prescription within 14 days of index MSK consultation (online supplemental appendix 5).

### MSK-HQ outcome

At baseline, participants from the most deprived neighbourhoods had lower mean MSK-HQ scores (poorer musculoskeletal health) than patients from the least deprived neighbourhoods (unadjusted mean difference: 5.08; 95% CI 3.28 to 6.88) (table 2). This difference reduced but remained statistically significant after adjustment for sociodemographic and survey covariates (adjusted mean difference: 4.25; 2.44–6.07). The baseline difference substantially reduced after further controlling for clinical case-mix variables (adjusted mean difference: 1.39; −0.35 to 3.13). Further adjustment for practice-level covariates had little further effect (1.30 (−0.45 to 3.06)).

**Table 2:** Association of deprivation with MSK-HQ score and interaction with response wave

		Model			
		β* (95% CI)	β† (95% CI)	β‡ (95% CI)	β§ (95% CI)
Deprivation group	IMDq1 (most)	Ref	Ref	Ref	Ref
	IMDq2	2.88 (1.31 to 4.45)	2.47 (0.90 to 4.04)	0.97 (−0.54 to 2.48)	0.85 (−0.67 to 2.37)
	IMDq3	3.74 (2.21 to 5.27)	2.99 (1.45 to 4.54)	0.66 (−0.81 to 2.12)	0.58 (−0.89 to 2.06)
	IMDq4	4.58 (2.95 to 6.22)	3.68 (2.03 to 5.34)	1.97 (0.39 to 3.55)	1.86 (0.27 to 3.45)
	IMDq5 (least)	5.08 (3.28 to 6.88)	4.25 (2.44 to 6.07)	1.39 (−0.35 to 3.13)	1.30 (−0.45 to 3.06)
Wave	Baseline	Ref	Ref	Ref	Ref
	3 month	4.07 (2.48 to 5.66)	3.82 (2.23 to 5.40)	4.01 (2.50 to 5.51)	4.00 (2.49 to 5.51)
	6 month	4.62 (2.98 to 6.26)	4.38 (2.74 to 6.01)	5.19 (3.65 to 6.72)	5.18 (3.64 to 6.72)
Wave x deprivation	3-month IMDq2	−0.21 (−2.66 to 2.24)	−0.23 (−2.67 to 2.22)	0.26 (−2.07 to 2.59)	0.26 (−2.07 to 2.59)
	3-month IMDq3	1.69 (−0.70 to 4.09)	1.79 (−0.61 to 4.18)	1.97 (−0.28 to 4.22)	1.97 (−0.28 to 4.22)
	3-month IMDq4	0.22 (−2.30 to 2.75)	0.50 (−2.03 to 3.03)	0.29 (−2.07 to 2.65)	0.29 (−2.07 to 2.65)
	3-month IMDq5	1.39 (−1.36 to 4.14)	1.45 (−1.29 to 4.20)	1.33 (−1.26 to 3.93)	1.33 (−1.26 to 3.92)
	6-month IMDq2	0.65 (−1.83 to 3.13)	0.70 (−1.78 to 3.18)	0.54 (−1.80 to 2.89)	0.54 (−1.81 to 2.88)
	6-month IMDq3	1.95 (−0.48 to 4.39)	2.08 (−0.35 to 4.52)	1.53 (−0.74 to 3.81)	1.54 (−0.73 to 3.82)
	6-month IMDq4	0.93 (−1.63 to 3.49)	1.07 (−1.50 to 3.63)	0.45 (−1.93 to 2.84)	0.45 (−1.94 to 2.83)
6-month IMDq5		2.32 (−0.49 to 5.14)	2.42 (−0.39 to 5.23)	1.94 (−0.70 to 4.58)	1.94 (−0.70 to 4.58)
Variance partition (practice level)		0.01	0.01	0.02	0.02

* Unadjusted.

† Adjusted for sociodemographic and survey-related covariates.

‡ Further adjusted for clinical case-mix covariates.

§ Further adjusted for practice-level covariates.

IMD, Index of Multiple Deprivation; IMDq1–q5, IMD grouped by quintile value, most deprived to least deprived; MSK-HQ, Musculoskeletal Health Questionnaire.

**Figure 2:**
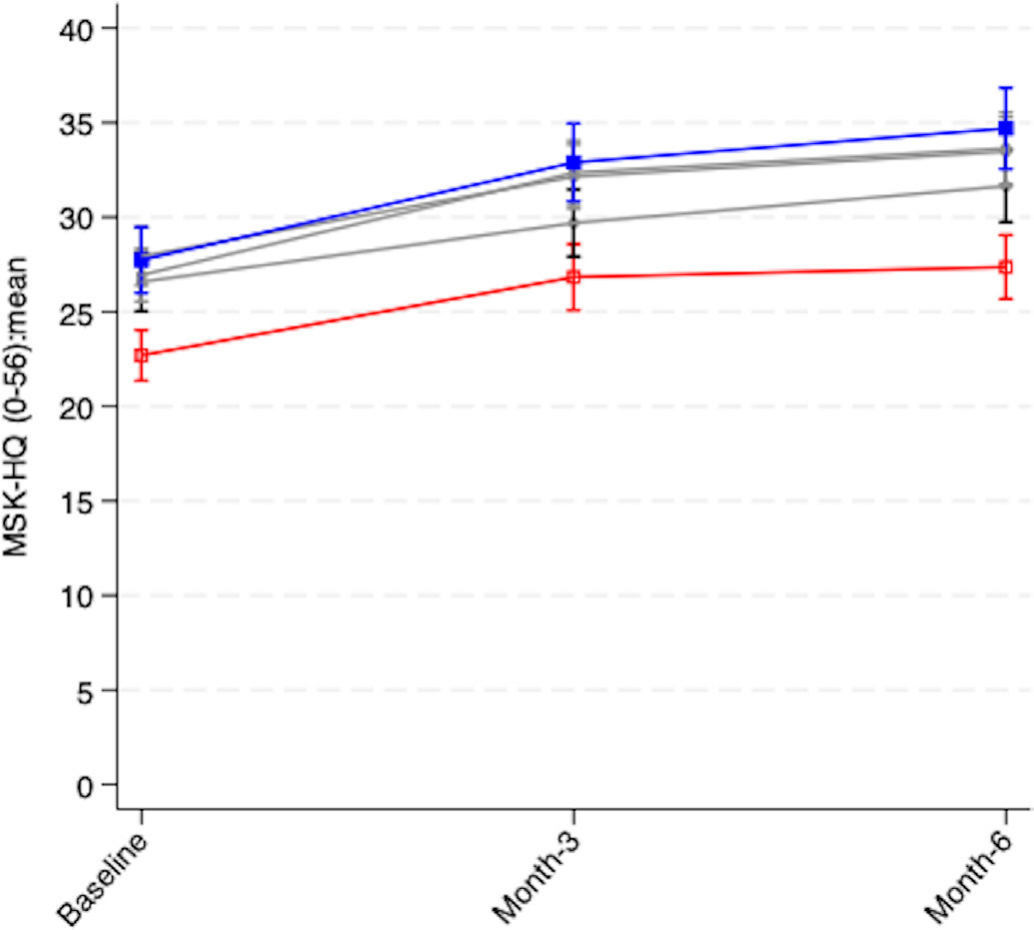
Unadjusted MSK-HQ mean scores, by deprivation (Index of Multiple Deprivation quintile) (red line=most deprived quintile; blue line=least deprived quintile). MSK-HQ, Musculoskeletal Health Questionnaire.

Overall participants’ MSK-HQ scores improved by a mean of 4.62 points (95% CI 2.98 to 6.26) by 6 months but the gap between deprivation groups did not narrow. Instead, those in the least deprived neighbourhoods improved by an additional (unadjusted) mean of 2.32 points (95% CI −0.49 to 5.14) compared with the most deprived group, and this difference remained consistent although not statistically significant across all models (figure 2).

Adjusting for practice-level staffing and musculoskeletal consultation rate did not affect the observed differences in MSK-HQ outcome between deprivation groups. Prior to the inclusion of any covariates, 7% of the variation in MSK-HQ score lay between practices, and this reduced to 1% when including deprivation and remained consistent on inclusion of the other covariates (table 2).

### Dissatisfaction with consultation

Only 6.4% of respondents reported dissatisfaction with their consultation (table 1). Those in the most deprived areas were more likely to report dissatisfaction, although this was not statistically significant (least versus most deprived unadjusted OR 0.64; 95% CI 0.34 to 1.19) and the association reduced on adjustment for sociodemographic and survey-related covariates (table 3).

**Table 3:** Association of deprivation with dissatisfaction with consultation and opioid prescription

	OR* (95% CI)	OR† (95% CI)	OR‡ (95% CI)	OR§ (95% CI)
Dissatisfaction with consultation				
Deprivation group				
IMDq1 (most)	Ref	Ref	Ref	Ref
IMDq2	0.68 (0.40 to 1.14)	0.84 (0.49 to 1.42)	0.93 (0.51 to 1.68)	0.97 (0.53 to 1.77)
IMDq3	0.69 (0.42 to 1.15)	0.94 (0.56 to 1.59)	0.90 (0.49 to 1.64)	0.92 (0.50 to 1.70)
IMDq4	0.43 (0.23 to 0.82)	0.60 (0.32 to 1.15)	0.46 (0.21 to 1.02)	0.47 (0.21 to 1.04)
IMDq5 (least)	0.64 (0.34 to 1.19)	0.89 (0.47 to 1.68)	0.92 (0.45 to 1.90)	0.99 (0.47 to 2.08)
Variance partition (practice level)	0.01	0.003	0.02	0.02
Opioid prescription¶				
Deprivation group				
IMDq1 (most)	Ref	Ref	Ref	Ref
IMDq2	0.86 (0.63 to 1.16)	0.83 (0.61 to 1.13)	0.96 (0.67 to 1.36)	0.95 (0.67 to 1.36)
IMDq3	0.99 (0.74 to 1.33)	0.94 (0.69 to 1.29)	1.09 (0.77 to 1.54)	1.10 (0.78 to 1.56)
IMDq4	0.77 (0.55 to 1.07)	0.73 (0.52 to 1.02)	0.80 (0.55 to 1.18)	0.81 (0.55 to 1.19)
IMDq5 (least)	0.58 (0.39 to 0.85)	0.54 (0.37 to 0.81)	0.65 (0.42 to 1.02)	0.69 (0.44 to 1.08)
Variance partition (practice level)	0.01	0.01	0.02	0.01

* Unadjusted.

† Adjusted for sociodemographic and survey-related covariates.

‡ Further adjusted for clinical case-mix covariates.

§ Further adjusted for practice-level covariates.

¶ Opioid prescription within 14 days of index consultation.

IMD, Index of Multiple Deprivation; IMDq1–q5, IMD grouped by quintile value; most deprived to least deprived.

### Opioid prescription

26% of participants received an opioid prescription within 14 days of their index musculoskeletal pain consultation (table 1). This varied from 30% in the most deprived neighbourhoods to 19% in the least deprived (unadjusted OR 0.58, 95% CI: 0.39 to 0.85, table 3). The strength of this association remained after adjustment for sociodemographic and survey-related covariates (adjusted OR 0.54; 95% CI 0.37 to 0.81) but weakened slightly after further adjustment for clinical case-mix covariates (adjusted OR 0.65; 0.42 to 1.02) and for practice-level covariates (adjusted OR 0.69; 0.44 to 1.08).

### Sensitivity analysis

Imputed analysis gave similar patterns as the main analysis for the associations between deprivation and MSK-HQ outcome, dissatisfaction with care and opioid prescription (online supplemental appendix 5), suggesting findings were not highly sensitive to missing data among participants.

## Discussion

We found substantial inequalities by socioeconomic deprivation in the severity and complexity of problems at, or around, the time of consultation among adults consulting GP for a musculoskeletal pain condition. The inequality gap in musculoskeletal health did not decrease over the following 6 months after consultation, but showed a small statistically non-significant increase, a finding which persisted after case-mix adjustment. Patients from more deprived areas were more likely to be prescribed opioid analgesia within 14 days of their index consultation, although differences reduced somewhat after case-mix adjustment.

Our multicentre prospective cohort study included GPs from across the spectrum of socioeconomic deprivation and from every PCN within a defined location and collected and linked individual-level data from EHRs, patient-report and public sources on neighbourhood and practice characteristics. The IMD, which we used to explore outcome inequalities, was recently endorsed as ‘a reasonable proxy for targeting communities with the greatest need’ in a national inquiry into musculoskeletal health inequalities.^5^ However, we acknowledge that an individual-level measure of socioeconomic position (eg, educational attainment, occupational class) could be more sensitive^24^; something our PAG felt was important.

A potential limitation of this study is that only 15% of all potentially eligible patients took part in the study and completed the baseline survey. Selective non-participation by deprivation—for example, a stronger ‘healthy volunteer’ effect among affluent patients—would result in an overestimate of outcome inequalities. Our PAG advised on ways of making study participation more accessible, several of which we incorporated, including keeping the study open for longer for practices based on more deprived areas, offering a mailed pen-and-paper self-complete option, letting participants know that close family friends/relatives could help with questionnaire completion if needed, minimising questionnaire length, using a logical order of questions. Selective loss to follow-up is also a threat to validity, with loss to follow-up greatest among participants from the most deprived neighbourhoods. After multiple imputation, findings were still consistent with those from complete-case analysis, although this assumes data missing at random given the covariates included in our model. Given high levels of missing data for outcomes, our findings should be treated as hypothesis-generating rather than definitive.^25^ Second, while the choice of case-mix adjustment factors was informed by a recent evidence synthesis and recommendations,^26 27^ we applied the same case-mix adjustment to patient-reported outcome, opioid prescription and patient experience. Future studies could explore the need for outcome-specific case-mix adjustment. Third, ‘baseline’ measurements were obtained typically 4–11 days after the index consultation. Patients can improve within such a timeframe,^28^ meaning that our ‘baseline’ measurements might over-estimate differences in MSK-HQ between more and less deprived patients at the point of care, while also underestimating improvements after the point of care. Participants in the current study did show markedly less improvement between ‘baseline’ and follow-up than previous pre-COVID studies in primary, community, workplace and secondary care settings (mean change in MSK-HQ score=5 points vs 9–10 points).^29^,^32^ However, ‘baseline’ values for MSK-HQ in the current study were also worse than those seen in most of these previous studies. The reasons for the poor level of improvement in MSK-HQ scores in the current study remain unclear. Fourth, only a small minority (6%) of patients in our study expressed dissatisfaction with the initial consultation based on our prespecified measure. We were therefore unable to estimate with precision any differences in patient experience by deprivation using this measure. We also note that long-term opioid analgesic prescription would have been a more useful indicator of care quality than a single opioid analgesic prescription. Fifth, our study was not sufficiently large to include practitioner as a distinct level within our multilevel analyses. Previous studies suggest that while variance in patient experience and outcomes is largely attributable to patient-level factors,^33^ differences between practitioners may be more important than differences between practices.^34^ Finally, our study did not restrict entry to those presenting with a first or new episode or to a specific musculoskeletal pain condition. Our findings relate to the heterogeneous population of new and ongoing patients presenting with a variety of musculoskeletal pain conditions in primary care.

To our knowledge, there are no directly comparable UK observational studies of outcome inequalities for musculoskeletal pain in primary care. Recent studies in Denmark and Sweden of community-based group-based osteoarthritis management programmes and digital self-management interventions consistently show similar socioeconomic inequalities (by educational attainment, income or country of birth/citizenship) in pain, function, disability and quality of life at the point of presentation.^35^,^37^ However, they have produced conflicting findings on whether inequalities widen or narrow following treatment, particularly over longer-term follow-up. Are the between-group differences observed in this study clinically important? While a within-person minimal important change of 5.5 points for MSK-HQ has been proposed,^32^ there is currently no established threshold for a clinically meaningful between-group difference in clinical trials. In a recent trial (ISRCTN38924614), we proposed a value of 3.6 points (which was equivalent to an effect size of 0.3). The mean between-group difference in MSK-HQ scores between the most and least deprived patients in the current study exceeds this, but it reduces after accounting for differences in clinical case-mix. Any widening in the inequality gap at follow-up is less than this value and therefore is not statistically significant and below a value that might indicate a clinically meaningful difference in the effectiveness of treatment for those from more deprived neighbourhoods.

Musculoskeletal pain constitutes a significant proportion of GP workload and population health burden. We found marked socioeconomic inequalities in the severity, complexity and prognosis of musculoskeletal pain at the time of presenting to primary care. It is already well-established that GP practices serving more deprived areas have fewer GPs^38^ and shorter consultations.^39^ Our findings reinforce the view that care follows a Disproportionate Care Law in which health inequalities are not reduced. This situation is likely to have been further exacerbated post COVID with longer waiting lists for elective surgery for conditions associated with long-term MSK pain. Future studies should continue to explore practitioner or service characteristics that promote equitable outcomes and quality of care, building on the action framework of Gkiouleka and colleagues.^40^ This includes critical research on equity impacts of additional primary care workforce roles, including first contact physiotherapists.^41^ However, marked inequalities in musculoskeletal symptoms, disability and chronicity are most likely already present at the point of consulting, suggesting that at least as much attention should be directed to timely, equitable access and actions earlier in the lifecourse at determinants and interventions beyond healthcare settings.

## Supplementary material

10.1136/bmjopen-2024-095132 online supplemental file 1

10.1136/bmjopen-2024-095132 online supplemental file 2

10.1136/bmjopen-2024-095132 online supplemental file 3

10.1136/bmjopen-2024-095132 online supplemental file 4

10.1136/bmjopen-2024-095132 online supplemental file 5

10.1136/bmjopen-2024-095132 online supplemental file 6

10.1136/bmjopen-2024-095132 online supplemental file 7

## Data Availability

Data are available upon reasonable request.
