# Supplementary material for: Socioeconomic inequalities in outcomes, experiences and treatment among adults consulting primary care for a musculoskeletal pain condition: a prospective cohort study
Source: BMJ Open. 2025 Jul 15;15(7):e095132. doi: 10.1136/bmjopen-2024-095132 (PMC12265837; doi:10.1136/bmjopen-2024-095132)
Supplement: online supplemental file 1 [file bmjopen-15-7-s001.pdf]

Full title: "Real world" pain outcomes and experiences of care (MIDAS-GP)

Short title: MIDAS-GP [Patient facing name: MIDAS-GP Study]

PROTOCOL VERSION NUMBER: v2.0 **ISSUE DATE:** 15-JAN-2022

RESEARCH REFERENCE NUMBERS

**IRAS Number:**	292109
**ISRCTN Number:**	ISRCTN18132064
**Sponsor Number:**	RG-0327-21
**Funder Number:**	OBF/43990

VERSION CONTROL

Version	Issue date	Reasons for amendments; additional changes
1.0	05Jul2021	Version 1

SIGNATURE PAGE

For Keele University sponsored studies, the sponsor will confirm approval of the protocol by signing the IRAS form and therefore a signature on the protocol is not required. The sponsor must be notified of all amendments to the protocol, both substantial and non-substantial. Review of amendments by the sponsor will act as the confirmation that the sponsor confirms approval of the amended protocol.

The undersigned confirm that the following protocol has been agreed and accepted and that the Chief Investigator agrees to conduct the research in compliance with the approved protocol, GCP guidelines, the Sponsor's SOPs, and other regulatory requirements as amended.

I agree to ensure that the confidential information contained in this document will not be used for any other purpose other than the evaluation or conduct of the clinical investigation without the prior written consent of the Sponsor

I also confirm that I will make the findings of the study publicly available through publication or other dissemination tools without any unnecessary delay and that an honest accurate and transparent account of the study will be given; and that any discrepancies from the study as planned in this protocol will be explained.

Chief Investigator:

Signature: Date:

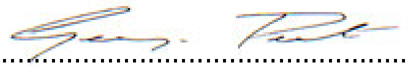

.... 14/06/2021

Name (please print):

Prof George Peat

....

Sponsor statement:

Where Keele University takes on the sponsor role for protocol development oversight, the signing of the IRAS form by the Sponsor will serve as confirmation of approval of this protocol.

LIST OF CONTENTS

SIGNATURE PAGE 3
LIST OF CONTENTS 4
LIST OF ABBREVIATIONS 6
KEY STUDY CONTACTS 7
STUDY SUMMARY 9
PLAIN ENGLISH SUMMARY 10
SUMMARY STUDY FLOW CHART 11
1 BACKGROUND AND RATIONALE 12
2 AIMS AND OBJECTIVES 12
2.1. Primary objectives 13
2.2. Secondary objectives 13
3 DESIGN 14
4 SETTING 14
5 ELIGIBILITY CRITERIA 14
5.1. For general practices 14
5.2. For participants 14
6 STUDY PROCEDURES 15
6.1. Overview 15
6.2. Strategies to improve inclusion and reduce bias 15
6.3. Patient-reported outcomes/experiences subcohort 17
6.3.1. Patient identification and recruitment 17
6.3.2. Informed consent 18
6.3.3. Data collection 18
6.3.4. Data linkage and extraction 20
6.3.5. Withdrawal criteria 21
6.4. EHR-only processes of care 21
6.5. Risk Mitigation 21
6.6. End of study 21
7 STATISTICS AND DATA ANALYSIS 22
7.1. Sample size calculation 22
7.1.1. Patient self-reported outcomes (primary objective 2.1.1.) 22
7.1.2. EHR-only processes of care outcomes (primary objective 2.1.2) 23
7.2. Planned recruitment rate 23
7.3. Statistical analysis plan 23
7.3.1. Patient reported outcomes and experiences 23
7.3.1.1. Summary of baseline data and flow of patients 23
7.3.1.2 Primary outcomes analysis 24
7.3.1.3 Secondary outcomes analysis 24

7.3.1.4 Sensitivity analysis 24
7.3.2 EHR-only processes of care outcomes 24
7.3.2.1 Summary of baseline data 24
7.3.2.2 Primary outcomes analysis 25
8 DATA HANDLING 25
8.1. Data collection tools and source document identification 25
8.2. Data handling and record keeping 26
8.3. Access to Data 26
8.4. Data Sharing Agreements 27
8.5. Archiving 27
9 MONITORING & AUDIT 27
9.1. Study Management 27
9.2. Independent Advisory Board 28
9.3. Study timeline 28
10 ETHICAL AND REGULATORY CONSIDERATIONS 28
10.1. Research Ethics Committee (REC) review & reports 28
10.2. Peer review 29
10.3. Public and Patient Involvement 29
10.4. Regulatory Compliance 30
10.5. Protocol compliance 30
10.6. Notification of Serious Breaches to GCP and/or the protocol 30
10.7. Data protection and patient confidentiality 30
10.8. Indemnity 31
10.9. Amendments 31
10.10. Access to the final dataset 31
11 DISSEMINATION POLICY 31
11.1. Dissemination plan 31
11.2. Authorship eligibility guidelines and any intended use of professional writers 32
12 REFERENCES 32

LIST OF ABBREVIATIONS

AB	Advisory Board
AE	Adverse Event
CI	Chief Investigator
CRF	Case Report Form
CRN	Clinical Research Network
CTU	Clinical Trials Unit
DMC	Data Monitoring Committee
EHR	Electronic Health Records
FCP	First Contact Practitioner
GCP	Good Clinical Practice
HCP	Health Care Professional
HRA	Health Research Authority
IRAS	Integrated Research Application System
ISF	Investigator Site File
ISRCTN	International Standard Randomised Controlled Trial Numbers
MSK	Musculoskeletal
NICE	The National Institute for Health and Care Excellence
NRS	Numerical Rating Scale
PCN	Primary Care Network
PI	Principal Investigator
PIC	Participant Identification Centre
PIS	Participant Information Sheet
PPIE	Patient and Public Involvement and Engagement
QA	Quality Assurance
QC	Quality Control
REC	Research Ethics Committee
RUG	Research User Group
SDV	Source Data Verification
SMF	Study Master File
SMG	Study Management Group
SMS	Short Messaging Service
SOP	Standard Operating Procedure
SSI	Site Specific Information

KEY STUDY CONTACTS

Chief Investigator	Professor George Peat<br>Primary Care Centre Versus Arthritis,<br>School of Medicine,<br>David Weatherall Building,<br>Keele University<br>Staffordshire, ST5 5BG<br>[redacted]
Associate Investigator	Dr Jonathan Hill<br>Primary Care Centre Versus Arthritis<br>School of Medicine<br>David Weatherall Building,<br>Keele University<br>Staffordshire, ST5 5BG<br>[redacted]
Sponsor	Head of Project Assurance,<br>Directorate of Research, Innovation and Engagement<br>Innovation Centre 2<br>Keele University<br>Staffordshire<br>ST5 5NH<br>[redacted]
Funder(s)	Nuffield Foundation<br>28 Bedford Square, London WC1B 3JS<br>Edmund McKiernan, Grants Coordinator<br>[redacted]
Study Management	Keele Clinical Trials Unit (CTU)<br>School of Medicine<br>David Weatherall Building<br>Keele University<br>Staffordshire, ST5 5BG<br>[redacted]
Key Protocol Contributors	[redacted]

	[redacted]
Lead Statistician	Professor Kelvin Jordan<br>Primary Care Centre Versus Arthritis,<br>School of Medicine,<br>David Weatherall Building,<br>Keele University<br>Staffordshire, ST5 5BG<br>[redacted]
Committees	**Study Management Group**<br>Peat (Chair), [redacted]<br>**Advisory Board**<br>[redacted]<br>**Patient Advisory Group**<br>[redacted]

STUDY SUMMARY

Study Title	"Real world" pain outcomes and experiences of care (MIDAS-GP)	
Internal Ref. Number (or short title)	MIDAS-GP<br>[Patient facing name: MIDAS-GP Study]	
Study Design	Observational Cohort Study	
Study Intervention (where applicable)	N/A	
Study Participants	Adults aged 18+ years consulting general practice for a common musculoskeletal pain condition	
Planned Sample Size	For patient-reported outcomes and experiences: 1,139 patients from 26 practices (completing a baseline questionnaire and consenting to further contact and individual record linkage)<br>For electronic health record (EHR)-only processes of care: 11,388+ patients from 26+ general practices (minimum of 150 patients per practice)	
Treatment duration	N/A	
Follow up duration	6 months (self-report outcomes); 12 months (EHR outcomes)	
Planned Study Period	July 2021 – Mar 2025	
	Objectives	Outcome Measures
Primary	To estimate the magnitude and direction of differences between potentially 'disadvantaged' and 'advantaged' groups of patients in their reported musculoskeletal health outcomes up to 6 months after consultation [patient-reported outcomes/experiences subcohort]	Expected main outcomes:<br>MSK-HQ score<br>Pain intensity (0-10 NRS)<br>Work productivity and activity impairment (WPAI)
	To estimate the magnitude of between-practice variation in processes of care for adults consulting with a MSK pain condition [EHR-only processes of care]	Expected main outcomes:<br>Practice-specific rates of primary care (re)consultation, secondary care referral, opioid prescribing, musculoskeletal imaging

PLAIN ENGLISH SUMMARY

AIM OF THE RESEARCH: To provide new research evidence to inform efforts to reduce variation in the outcomes and experiences of care between different groups of patients with musculoskeletal pain and between different general practices.

BACKGROUND: Painful musculoskeletal conditions like back pain and osteoarthritis cause more disability in the general population than any other health conditions. Poorer communities and individuals appear to be hardest hit. In order to have a suitably 'joined up' response to this challenge we need accurate and meaningful joined up information on musculoskeletal health, risk, and care in local populations. This is what our study will try to address.

DESIGN AND METHODS: We will invite adults who have recently consulted their general practice with a painful musculoskeletal condition to tell us about their condition and their care. People will have the option of doing this through an online questionnaire (with support over the telephone, if needed) or by pen-and-paper questionnaire. If people agree, we will contact them again with short questionnaires up to 6 months later to understand if they have got better. We will also ask them if we can link their questionnaire responses with information held in their medical records so that we can piece together the type of care people are receiving with the kind of problem they have and the outcome of their care. We want to involve at least 26 general practices across North Staffordshire and Stoke-on-Trent, particularly those serving more deprived and ethnically diverse areas. Despite our best efforts, we know that not all people will agree to take part. So, to get a proper overview, a second part of this study will look at the overall levels of prescribing painkillers, referrals to hospital specialists, and other measures of musculoskeletal care for each general practice.

PATIENT AND PUBLIC INVOLVEMENT: We have a dedicated Patient Advisory Group who have already been involved in designing this study. They have:

- stressed the importance of looking seriously at inequalities in health and care
- suggested ways of raising awareness, maintaining interest in the study, and making it easier for a wide range of people to take part
- looked carefully at the questionnaires and suggested ways of making it more relevant and easier to complete

We will continue to work with this Group to monitor how the study is going, what the findings mean, and how best to share them with participants, the public, and other groups and maximise our chances of this research making a real difference.

SHARING OUR FINDINGS: We will look to produce:

- Written summary reports, graphs and tables for participating GP practices and community musculoskeletal services
- Press releases, briefings, articles, and interviews for local radio and newspapers
- A study website, institutional websites, and post messages on social media including Twitter, YouTube video
- Publications (to include public co-authors) including full report, executive summary and plain English summary, peer-reviewed journals, and local NHS and research newsletters
- Presentations at high-profile scientific and health policy conferences: NHS Evidence, Society for Academic Primary Care, Chartered Society of Physiotherapy, Public Health England

SUMMARY STUDY FLOW CHART

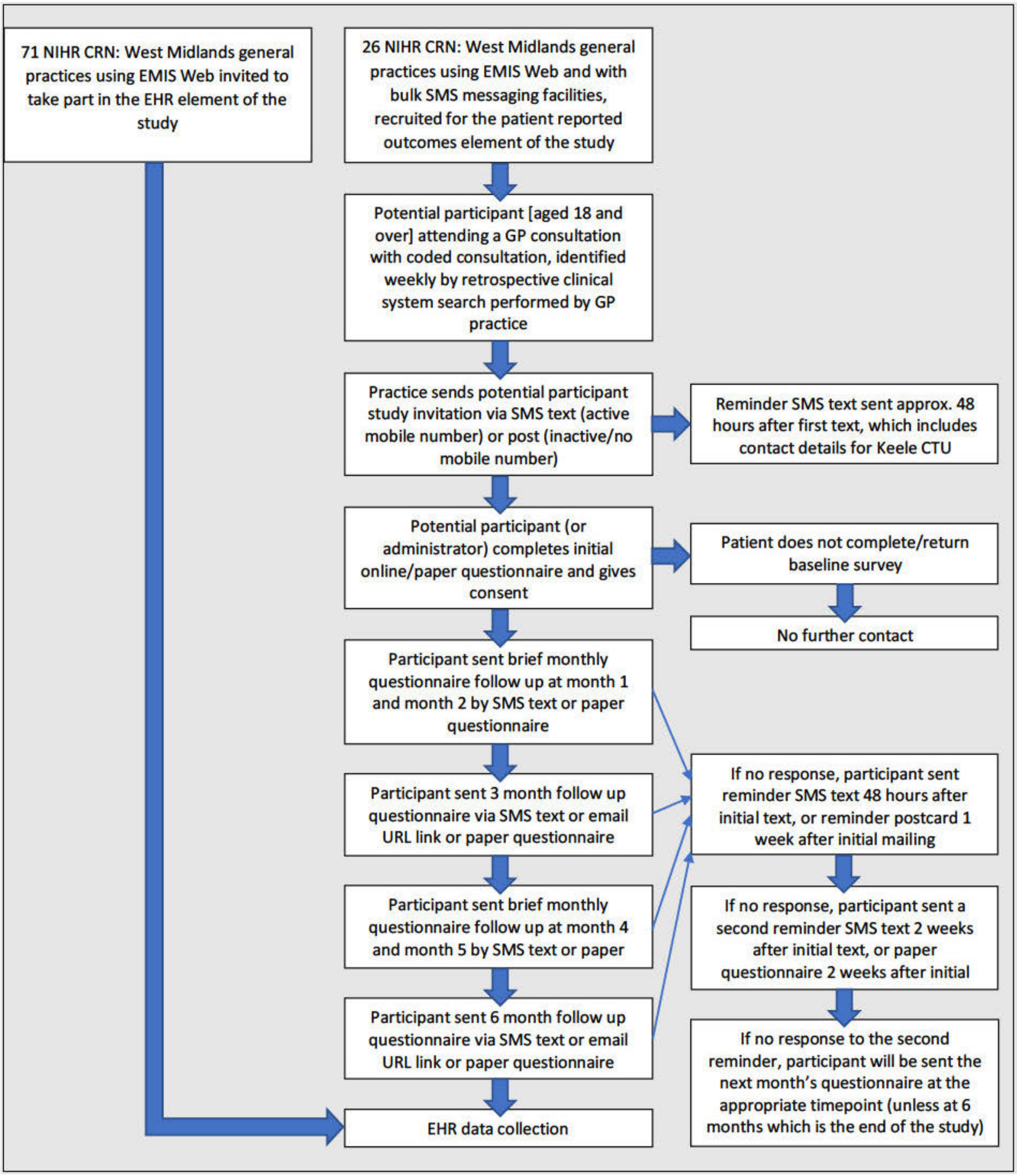

1 BACKGROUND AND RATIONALE

Musculoskeletal (MSK) conditions are the main drivers of non-communicable disease disability burden in most countries and regions worldwide.[1] In England, they account for an estimated 21% of total years lived with disability,[2] 6.2 million working days lost,[3] 12-14% of all primary care consultations in people aged 15 years and over,[4,5] and the third largest programme budget for NHS healthcare expenditure.[6] The need for better information for chronic disease surveillance of MSK conditions has been highlighted by the Chief Medical Officer[7] and in successive Global Burden of Disease reports for England.[8,9] Public Health England's 5-year strategy for musculoskeletal health (2019-2024) includes a commitment to "high quality, accessible data and intelligence tools to support surveillance and reduce unwarranted variation of musculoskeletal conditions across the population pathway."[10]

Our MIDAS programme of research, funded by the Nuffield Foundation and Versus Arthritis, seeks to develop and evaluate a place-based system for population musculoskeletal health intelligence across North Staffordshire and Stoke-on-Trent (Enriched data integration for population musculoskeletal health intelligence | Nuffield Foundation).

The current study – an observational cohort study of the episode outcomes of patients presenting to general practice with a common, painful MSK conditions - is one component of this programme. It addresses an important priority for the NHS: to better integrate data from different clinical settings within the musculoskeletal clinical pathway in order to inform system wide changes that can reduce the burden of common MSK conditions. To date, meaningful data collected from population level surveys, primary care electronic health records and community service providers have not been co-ordinated and linked together to inform public health and primary care policy making.

In order to link data together, we plan to conduct a prospective patient cohort of adults consulting a healthcare professional (predominantly GP or First Contact Practitioner (FCP)) situated within participating GP practices, for a painful MSK condition. The data collected will come from one of three sources:

(1) patient questionnaires: for measures of severity, impact, levels of knowledge, confidence, and understanding of their condition, experiences of care, and wider social determinants.

(2) electronic medical record data: for recorded care (e.g. prescriptions, referrals, imaging, Fit Notes, repeat GP visits) and comorbidities

(3) publicly available data on neighbourhood health, assets and deprivation and on healthcare service characteristics

By linking these sources of data we are seeking to create multi-level data on patient cohorts that enable us to better understand variations in, and determinants of, musculoskeletal outcomes in adults presenting to primary care with a common painful MSK condition. Our intention is for this study to impact on decisions about what information may be most useful and how it might be collected, linked, analysed, and disseminated within routine care.

2 AIMS AND OBJECTIVES

The **overall aim** of this prospective cohort study is to investigate variation and inequalities in patient-reported outcomes and experiences of care and the type of care received by adults presenting to general practice with a non-inflammatory musculoskeletal (MSK) pain condition. The study comprises two main components:

(a) Patient-reported outcomes/experiences: collection via online and offline surveys of patient-reported outcome and experience measures up to 6 months after consultation and, with individuals' consent, linkage to primary care electronic health record data and NHS Digital datasets and place-based information on wider determinants of health. While all eligible MSK consulters will be invited to participate in this component of the study, only a self-selected proportion are anticipated to take part in this study component.

(b) EHR-only processes of care: extraction and analysis of pseudonymised data from the primary care electronic health record on recorded processes of care for all eligible consecutive MSK consulters during the study period and prior to this. By descriptive analysis of record-level data on the total underlying target population this component will enable us to evaluate self-selection bias in the patient-reported outcomes/experiences subcohort, enable adequately powered investigation of variations between GP practices in recorded care, and extend our findings from a single snap-shot view in time.

2.1. Primary objectives

2.1.1. To estimate the magnitude and direction of differences between potentially 'disadvantaged' and 'advantaged' groups of patients in their reported MSK health outcomes up to 6 months after consultation [patient-reported outcomes/experiences subcohort]

2.1.2. To estimate the magnitude of between-practice variation in rates of primary care (re)consultation, secondary care referral, opioid prescribing, and musculoskeletal imaging for adults consulting with a MSK pain condition [EHR-only processes of care]

2.2. Secondary objectives

2.2.1. To estimate differences between potentially 'disadvantaged' and 'advantaged' groups of patients in their experiences of primary care MSK consultation [patient-reported outcomes/experiences subcohort]

2.2.3. To explore within-practice change in consultation prevalence and recorded management of MSK pain among adults presenting to general practice over time, including comparing current levels with those before COVID (i.e. pre-2020) [EHR-only processes of care]

2.2.4. To plot the flow of patients along different MSK care pathways and to define MSK service organisation characteristics for participating general practices and Primary Care Networks (PCNs) [patient-reported outcomes/experiences subcohort]

2.2.5. To produce new benchmarked data on processes and outcomes of care for MSK pain conditions at GP practice and PCN levels and to provide new insights into the credibility, validity, and persuasiveness of new visualisations of this MSK health intelligence and present these for feedback from key stakeholders

2.2.6. To explore the relationships between identified variations and inequalities in patient care, outcomes, and experiences and their association with wider determinants and organisational characteristics

2.2.7. To test case-mix adjustment methods for identifying outliers for recorded processes of care

2.2.8. To evaluate patterns of non-response and non-participation and their implications for bias in the above estimates

3 DESIGN

Observational cohort study with 6-month follow-up for self-reported outcomes and 12-month follow-up for EHR outcomes.

4 SETTING

General practices in North Staffordshire and Stoke-on-Trent, with the option to extend to additional general practices in the West Midlands (e.g. Wolverhampton).

5 ELIGIBILITY CRITERIA

5.1. For general practices

Inclusion	Exclusion
Located in North Staffordshire or Stoke-on-Trent, (wider Staffordshire / West Midlands if needed)[a]	
Uses compatible IT system (e.g. EMIS)	
Uses compatible SMS messaging service (e.g. MJog)[b]	
Willing and able to undertake regular anonymised medical record audits of MSK consultations during the study period	
[a] see section 4 above for circumstances under which widening GP recruitment beyond North Staffordshire & Stoke-on-Trent will be considered<br>[b] not required for EHR-only processes of care<br>MSK Musculoskeletal; SMS Short message service	

5.2. For participants

Inclusion	Exclusion
Patients aged 18 years and above	Inflammatory musculoskeletal disease[c]
Registered with a participating general practice during the study period	Has indicated in the record that they do not consent to be approached about research studies
Consulting any primary healthcare professional in the general practice for a painful, non-inflammatory musculoskeletal disorder[a] during the study period	

Able to provide informed consent[b]	
[a] according to predefined SNOMED code lists<br>[b] patient-report survey component only<br>[c] according to predefined SNOMED code lists retrospectively examined over the previous 3 years<br>NB participating general practices will have the option of screening and excluding potentially eligible MSK consulters whom they deem to be vulnerable or inappropriate to invite for patient-reported outcomes/experiences due to health reasons (e.g. severe mental health condition, significant cognitive impairment, recent diagnosis of terminal illness, nearing end of life)	

6 STUDY PROCEDURES

6.1. Overview

Potential participants will be those consulting a participating general practice for a relevant MSK pain related problem during the study period. Relevant MSK pain-related consultations will be identified using a pre-specified SNOMED codelist. Eligible patients with an active mobile phone number will be invited via SMS text sent from the practice computer system to complete a web-based questionnaire, based on a secure server at Keele University. Patients without an active mobile telephone number at the practice will be posted a questionnaire for pen-and-paper completion via DOCMAIL, a secure remote document compilation, print and mailing solution. Follow-up questionnaires will be sent by Keele CTU at 3 and 6 months, with a brief questionnaire sent in the intervening months (SMS text or postal). All participants will be asked for informed consent to link their questionnaire responses to primary care electronic health record and healthcare datasets (e.g. hospital visits) held by NHS Digital. This "patient-reported outcomes/experiences subcohort", providing individual-level, linked patient-reported and health record data, will be used to answer questions on variations in are outcomes and experiences of care. Separately, pseudonymised information from primary care electronic health records will also be sought from all eligible patients consulting a participating practice with a relevant MSK pain condition during the study period and across the previous five years. These will be analysed and presented at the level of GP practice and PCN to add a wider, longer-term view of between-practice and between-PCN variation in care.

6.2. Strategies to improve inclusion and reduce bias

Participation rates in cohort studies have been declining over several years raising concerns over inefficiency and the potential for selection bias. The use of web-based data collection is increasingly pursued as a low-cost solution to the former problem but may have lower response rates[11] and is still susceptible to selective participation. Internet access in UK households continues to increase year-on-year (96% in 2020[12]) but people most likely to be 'digitally excluded' are: older people, people in lower income groups, people without a job, people in social housing, people with disabilities, people with fewer educational qualifications, people living in rural areas, homeless people, people whose first language is not English.[13] These groups may be 'disadvantaged' or 'under-served'[14] and have more complex health needs and poorer outcomes.

The following strategies to improve inclusion and reduce bias are informed by discussions with our Participant Advisory Group, previous synthesis of evidence on the effectiveness of different strategies for survey completion, NIHR INCLUDE[14] and INCLUDE Ethnicity[15] frameworks, previous

experience within the research team, and considerations over what is feasible and affordable for our study.

- Sampling of general practices from across all PCNs in North Staffordshire and Stoke-on-Trent. We will try to over-sample practices in the most deprived neighbourhoods and with a higher proportion of Black, Asian, and minority ethnic patients. To reach a sufficiently diverse population, we will also consider offering study participation to general practices in South Staffordshire and the West Midlands (e.g. Wolverhampton).

- Use of a mixed-mode approach in which those unable or unwilling to complete online questionnaires are offered conventional pen-and-paper self-complete questionnaires.[16] We are not using an explicit ‘push-to-web’ design (i.e. where web completion is offered as the only mode and alternatives like paper completion are withheld until later on, e.g. for non-respondents).[17]

- The offer of telephone support for questionnaire completion; explicit reassurance that family members can assist (but not substitute) in questionnaire completion for the intended respondent

- The offer of access to a translation service via telephone for study information and questionnaire completion

- Keeping questionnaire length to a minimum[11,18]; presenting questions in a logical order; minimising cognitive burden of questions; explaining where possible the purpose of questions

- Raising awareness of the survey among the practice and registered patient population prior to going live; using reminders to encourage questionnaire completion[11,18]

- Collection of brief information in the questionnaire on important social characteristics (e.g. date of birth, sex, ethnicity, occupational class, financial strain) to help understand participation, care, and outcomes in under-served groups

- Using pseudoanonymised extracts from the GP clinical system to describe the characteristics of those invited to participate (e.g. Practice Code, Practice Name, Age, Sex, Lower Super Output Area (from postcode), Ethnicity, Method of Invite), as well as the characteristics and patterns of care of the total eligible population of MSK consulters to enable evaluation and possible modelling of selective participation

Those who participate in the study and return either a paper or online questionnaire will be given the opportunity to select a local charity that they would like us to make a donation to. At the end of the initial, 3-month and 6-month questionnaires, participants will be given a choice of four local charities to give to. For every returned baseline, 3-month and 6-month questionnaire £1 will be given to the charity specified by the participant.

6.3. Patient-reported outcomes/experiences subcohort

6.3.1. Patient identification and recruitment

Consecutive eligible patients consulting a participating general practice during the study period will be invited to take part.

Identification of eligible consultations

When a pre-defined MSK pain–related SNOMED code (symptom or diagnostic code) is entered into the primary care electronic health record, a tag related to the research project will be added to the patient record. Practice staff, supported by the NIHR Clinical Research Network (CRN) where possible, will regularly (typically weekly) run an automated search of their general practice's medical record to identify consulters with a relevant tagged MSK record from the previous week. The automated search includes a screen of the identified patient list to exclude ineligible patients according to exclusion criteria in protocol. GP practices will also have the option of performing a manual screening of the identified list.

Patient invitation

Using the general practice's existing system for SMS texts to patients identified eligible consulters who have an active mobile phone number on the practice record will be sent an SMS text invitation from the general practice to take part in the research. Identified eligible consulters who do not have an active mobile phone number will be sent an invitation pack via Docmail. The invitation pack will include an invite letter (containing URL link to enable online questionnaire completion if so desired), baseline questionnaire with consent form, return pre-paid envelope and a Participant Information Sheet containing the contact details for Keele CTU should the patient wish to seek more information about the study or seek help to complete the questionnaire.

All potential participants receiving an SMS text invitation will be sent a reminder text approximately 48 hours after the initial text, which will include information on how to contact Keele CTU if they are unable to complete the questionnaire online.

Potential participants who do not respond will receive no further contact and will not be re-invited from the same practice, for example, if they revisit their GP within the study recruitment period.

When potential participants arrive at the online questionnaire, they will be provided with the opportunity to read a Participant Information Sheet and view an information video about the study.

The online questionnaire will be hosted by Keele University, with the option of telephone supported completion from a Keele CTU administrator or translation service. Respondents will be sent a follow-up questionnaire at 3 and 6 months after their initial consultation with a brief questionnaire sent in the intervening months' to capture pain intensity.

Participating GP practice will send an Excel file to the study team on a regular basis (approximately weekly), via NHS.net secure email. This file will contain the Organisational Code, NHS number, anonymised identifier, invite date and method of invitation for each patient sent an invitation to take part in the study. This file will be imported into the Study Management Application by a study administrator. When questionnaires are received into the CTU (online or paper) the information from their consent to take part in the study will be matched to the information from the import file, this will also ensure that the correct patient is taking part in the study.

6.3.2. *Informed consent*

Potential participants in the patient-reported outcomes/experience subcohort will be asked for their consent to retain and analyse their questionnaire responses, to be contacted again in future for follow-up questionnaires for this study, to access relevant information from their medical records held at their general practice and by NHS Digital and link this to their questionnaire responses.

In both online and pen-and-paper questionnaire formats, informed consent from willing patients will be obtained at the end of baseline questionnaire completion. For the online questionnaire this will be e-consent through the secure web-based interface hosted by Keele University; for the pen-and-paper it will be written consent; for those opting for telephone-supported completion of the questionnaire, the administrator will obtain verbal consent. Consent is being sought after patients have completed the online questionnaire to ensure that patients know what data they are providing before agreeing to it, this is in line with how patients will be completing the paper questionnaire. If a patient stops the initial online questionnaire part way through and changes their mind before they consent, the data they have provided will be destroyed.

The consent section will include mandatory statements that potential participants must confirm that they agree to in order to participate in the study.

As part of the baseline questionnaire, a minimal set of participant identifiable data will be collected in order to ensure an individual is correctly identified and that the right participant is identifiable for follow-up questionnaire completion. Both consent formats cannot be altered by the participant and will be dated either automatically by the web-based system for online completion or by the patient for manual completion. If the consent date is missing from the pen-and-paper consent form, the date the questionnaire was received will be added upon entry to the database by an administrator. If the patients' signature is missing from the pen-and-paper consent form, a completed consent form will be sought by following the incomplete consent procedure. Prior to seeking consent to study participation, all potential participants will have had the opportunity to access the Participant Information Sheet and contact a member of the study team. Patients who contact Keele CTU and provide consent over the telephone will be posted a hard copy of the Participant Information Sheet.

If potential participants decline the e-consent to take part in the study they will receive an onscreen notification thanking them for considering participation and explaining that they will not be contacted further. Those who do not consent to take part in the study will be notified all of their responses to the baseline questionnaire will be deleted.

We will also ask patients to provide either a) their mobile telephone number or email address (online questionnaire), b) their postal address and telephone contact number (postal questionnaire and from those completing over the telephone) for them to receive their follow-up questionnaires.

Patients completing the online consent will be given the option to obtain a copy of the consent form by emailing the study team.

6.3.3. *Data collection*

Table 1. Schedule of assessments for the patient-reported outcomes subcohort

	Timepoint (months)						
	0	+1	+2	+3	+4	+5	+6
MSK health							
Pain location	***x***						

Pain intensity (0-10 NRS) [19]	x	x	x	x	x	x	x
Pain days [20]	x			x			x
Pain interference [20]	x			x			x
Duration of problem	x						
Previous pain episodes	x						
Previous surgery	x						
MSK-HQ [21]	x			x			x
Patient global rating of change	x			x			x
Patient experience							
Overall experience [22]	x						
Communication [23]	x						
Needs met [22]	x						
Personalised plan	x						
Healthcare utilisation							
HCP/service use for MSK	x			x			x
(Self-)management for MSK	x			x			x
General health							
Height and weight	x						
Health literacy [24]	x						
Demographic/socioeconomic							
Age (date of birth)	x			x			x
Sex	x			x			x
Ethnicity [25]	x						
Perceived financial strain [26]	x						
Employment							
Employment status	x			x			x
Job title (current/most recent)	x						
WPAI [27]	x			x			x
**Administrative**							
NHS number	x						
GP practice	x						
HCP Health care professional; MSK-HQ Musculoskeletal Health Questionnaire; WPAI Work Productivity and Activity Impairment (modified version)							

Baseline patient-reported questionnaire

Table 1 lists the patient reported outcome and experience measures to be collected by patient questionnaires. The content of the questionnaire was informed by a cross-mapping of recommended indicator sets [e.g. 28], and measures used in national surveys (e.g. GP Patient Survey, Health Survey for England, FCP evaluation) and previous research studies in the same population and setting. For evaluating social inequalities we used the PROGRESS-Plus framework[29,30] to inform our choice of individual- and area-level social stratifiers.

Brief monthly patient-reported questionnaires

At months 1, 2, 4, and 5 following the index consultation the patient will be asked one question to report their pain intensity either using SMS text or postal questionnaire. Keele CTU has experience of using 2-way SMS text messages to collect outcome data in previous trials (Foster et al 2017,

Campbell et al 2016). The software development team within the CTU will support this data collection and will make use of an in-house bespoke database-driven Web-based system to manage the sending and receiving of SMS text messages. The participant will respond to Keele CTU via the third-party SMS provider. The CTU system will poll the third-party SMS provider for returned SMS text messages. On processing of returned SMS text messages, the system will import these into the study database and process the response according to the business logic defined in the project. Interception of the original message by a third party would reveal only what the questions were that were being asked, whilst interception of the response would only yield an alphanumeric string.

A telephone number will be provided to enable participants to contact Keele CTU if they have queries relating to the SMS text messaging during office hours.

Follow-up patient-reported questionnaires

The questionnaire will collect follow-up outcomes at 3 and 6 months including MSK health, healthcare utilisation, and work productivity and activity impairment.

6.3.4. Data linkage and extraction

Primary care electronic health record

Information on comorbidities and MSK management, including those needed to address the primary objective, will be collected from the primary care electronic health record. This will include relevant prescription medications, referrals to other services (e.g., physiotherapy and secondary care specialists), referrals for investigations (e.g., radiographs, MRI/Computerized Tomography (CT) scans), sick certifications (fit notes), and further MSK-related primary care consultations.

NHS Digital datasets

Linkage to MSK-relevant hospital outpatient appointments, admissions, accident & emergency attendances (Hospital Episode Statistics) and diagnostic imaging (Diagnostic Imaging Dataset) outcomes held by NHS Digital will be sought through the Data Access Review Service (DARS).

Healthcare provider characteristics

Non-sensitive aggregate- and global-level data on general practices and MSK services (e.g. staffing levels, Quality and Outcomes Framework (QOF) performance) will be extracted from the freely available general practice workforce data (NHS Digital General Practice Data Hub), PHE National General Practice Profiles, and GP Patient Survey data. During site initiation visits with participating general practices we will clarify the current provision of selected recommended MSK services, e.g. First Contact Practitioner physiotherapists, vocational advice, stratified care for low back pain [31,32].

Neighbourhood characteristics and assets

Aggregate data on wider determinants of health (e.g. healthy diet, obesity, labour market, housing, built environment, journey time statistics) in local geographies (lower and middle super output areas, CCG, unitary authority) will be extracted from existing accessible sources (e.g. Strategic Health Asset Planning and Evaluation (SHAPE: health-related determinants); NOMIS (labour market statistics); Public Health England's Data Gateway and Local Health tools) and linked to individual-level datasets above to create multi-level data. This includes modelled estimates of the underlying population prevalence of MSK pain and disability from our previous PRELIM survey.

6.3.5. *Withdrawal criteria*

Patients can withdraw from the patient-reported outcomes subcohort at any time by contacting and informing Keele CTU by telephone, email or letter. Withdrawal will mean no further reminders or invitations to complete questionnaires will be sent. Any information provided up to the point the participant withdraws will be anonymised and retained unless the request is made for data to be destroyed.

6.4. EHR-only processes of care

For EHR-only processes of care objectives we will extract and analyse pseudonymised data from participating GP practices on relevant prescriptions (e.g. strong opioids), referrals, imaging, Fit Notes, and repeat GP visits among all eligible adult MSK consulters (defined in 5.2 above). Data extracted will be for the period July 2016 to Sep 2023. This retrospective period is required to provide a better understanding of changes over time in MSK care in participating GP practices (including whether between-practice differences in care are recent or more longstanding and pre-dating COVID; objective 2.2.3).

6.5. Risk Mitigation

For individual patients who consult a participating general practice with a MSK pain condition, the potential risks from being asked to complete initial, three and six-month follow-up questionnaires plus a text or brief postal questionnaire about pain intensity in the intervening months, are considered to be very low. All questionnaire mailings will be sent within an enclosed envelope and include a pre-paid reply envelope to ensure patient confidentiality at all times. Questions are on the severity and nature of pain and other symptoms such as sleep disturbance and are therefore not considered a risk beyond minor inconvenience.

To ensure that consenting participants in the patient-reported outcomes sub-cohort are appropriately linked to the medical record data we will use NHS number as the key link variable. Furthermore, to check that the individual concerned is completing the questionnaire we will collect name of GP practice and date of birth as further validation items.

To ensure high response rates to the questionnaire, sensitive items (as judged by the Patient Advisory Group) have been removed from the questionnaire.

General practices will receive payment for the additional time required to set-up the study and retrospective searches they perform.

6.6. End of study

The end of the study will be when the last participant completes the 6-month follow-up data collection process and all relevant medical record data have been collected.

7 STATISTICS AND DATA ANALYSIS

7.1. Sample size calculation

7.1.1. Patient self-reported outcomes (primary objective 2.1.1.)

We anticipate from previous studies and discussions with GP colleagues that the level of practice engagement may wain if the recruitment period extends much beyond 3 months.

The median registered population size of general practices in North Staffordshire & Stoke-on-Trent is 7300. Assuming 80% are aged 18 and over and an annual consultation prevalence for musculoskeletal pain of 15%[33], then there will be 876 patients consulting for musculoskeletal pain on average per practice each year. Across 26 practices (2 per PCN), there will be 5694 eligible patients presenting in the 3-month recruitment period. Assuming a 25% response at baseline and 50% at follow-up, then there will be 1424 baseline responders (1139 (80%) consenting to further contact and record linkages) and 569 responders at 6-month follow-up (**Figure 1**).

Figure 1. Estimated number of patients recruited and followed up for the patient-reported outcomes subcohort (NB 3-month period of recruitment per practice).

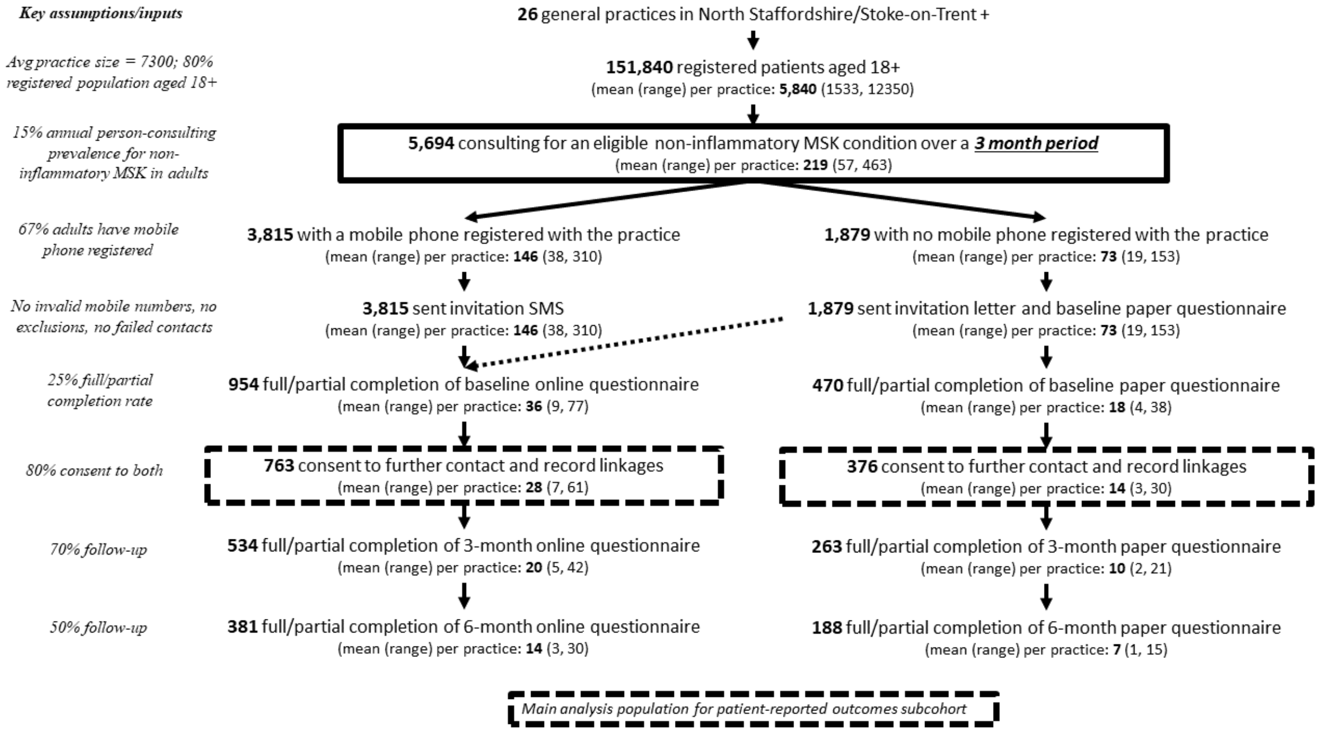

The baseline response of 1424 will allow us to detect a difference of 3 or more points on the MSK-HQ (assuming standard deviation of 10) with 90% power at the 5% significance level for groups defined by a dichotomous covariate with prevalence of 10% (for example ethnicity: black, Asian, and ethnic minority patients vs white).

569 responders at 6-month follow-up will also allow us to detect a difference on the follow-up MSK-HQ of 3 or more points (assuming standard deviation of 10), with 80% power at the 5% significance level, for groups defined by a dichotomous covariate. This is based on a covariate with a prevalence of 10%, 2 follow-up time points (3m and 6m), adjustment for baseline MSK-HQ score, and assumed correlations of 0.5 between the two follow-up scores and between the follow-up and baseline

scores.[34] We will, however, use repeated measures multilevel models to ensure all patients responding at baseline can be included in the analysis.

7.1.2. EHR-only processes of care outcomes (primary objective 2.1.2)

Our study population will be all adults consulting for a relevant musculoskeletal pain condition over the study period. From the estimates in 7.1.1., we expect an average of 219 patients per practice over a 3-month period. This exceeds the 150 patients recommended as the minimum needed per service for assessing variation between providers using, for example, funnel plots.[35] Smaller practices (list size<5,000) are likely to fall below this target of 150 adult MSK pain consulters over a 3-month period. We therefore intend to extract pseudonymised, record-based outcomes for eligible adults consulting for a MSK pain condition in each practice over 6-month intervals. This will enable practices with list sizes as small as 2500 to meet the target of 150 patients. For 26 practices, this yields a total sample size of roughly 11,388. However, for this component of the study, where the burden on general practices is very low, we will extend the invitation to participate to all general practices in North Staffordshire & Stoke-on-Trent using the EMIS clinical IT system and, if necessary, to other eligible and interested practices in the West Midlands.

7.2. Planned recruitment rate

The rate of patient recruitment for the patient reported outcomes per practice is detailed above. The overall rate of patient recruitment to this study will depend on when each practice begins patient recruitment. Due to practice circumstances, CTU and CRN capacity, we anticipate a staggered start for practices over a 3-month period (**Table 2**).

Table 2. Planned recruitment rate, by month

Month	1	2	3	4	5	6
No. of GP practices recruiting	8	17	26	18	9	-
Cumulative eligible adult MSK consulters	584	1825	3723	5037	5694	-
Cumulative no. of participants completing baseline questionnaire[a]	146	456	931	1259	1424	-
[a] Does not account for lag between consultation and completion of baseline questionnaire (est. average 2w)						

7.3. Statistical analysis plan

7.3.1. Patient reported outcomes and experiences

7.3.1.1. Summary of baseline data and flow of patients

We will determine the percentage of eligible patients responding at baseline and descriptively compare responders to all eligible patients consulting during the recruitment time period by age, gender, type of musculoskeletal pain, general practice and PCN.

We will report summary statistics (mean and standard deviation, median and interquartile range or frequencies and percentages as appropriate) for each baseline and follow-up measure, overall and stratified by age, gender, and type of musculoskeletal pain. We will also weight responses by age,

gender, and type of musculoskeletal pain distribution of eligible patients to assess the potential impact of non-response on our estimates.

Missing data within responders at each time point should be low but we will use multiple imputation if there are items with >5% missing data.

7.3.1.2 Primary outcomes analysis

We will report summary statistics (mean and standard deviation, median and interquartile range) for the MSK-HQ baseline, 3-month and 6-month score and its mean change over 3- months and 6-months overall and stratified by age, gender, and type of musculoskeletal pain. We will also determine the percentage of patients who achieve the minimal important change (MIC) for the MSK-HQ. This has been reported as 6 points, though varies by body region of pain. We will weight responses by age, gender, and type of musculoskeletal pain at baseline.

We will determine patient characteristics associated with baseline MSK-HQ score using multilevel linear regression models (patients nested within practices) including as covariates the key case-mix variables.

To assess inequalities in change in musculoskeletal health over the 6-months, we will use multilevel repeated measures linear regression models (follow-up measures nested within patients) to determine patients characteristics associated with MSK-HQ score at 3m and 6m, adjusting for baseline MSK-HQ score and the case-mix variables.

7.3.1.3 Secondary outcomes analysis

We will repeat the analyses described above for the percentage of patients achieving the minimal important change on the MSK-HQ (using multilevel repeated measures logistic regression models) and for the secondary outcomes, including pain interference, and work productivity (WPAI-modified version). For the monthly pain intensity outcome, our models will include all follow-up responses.

7.3.1.4 Sensitivity analysis

The advantage of using multilevel repeated measures models is that all baseline responders can be included in the analysis even if they do not respond at one or more follow-up points. However, for patients responding at baseline but not at 3 or 6 months, we will also use multiple imputation including baseline and monthly measures (where available) to impute 3- and 6-month follow-up MSK-HQ scores and repeat the analyses above.

7.3.2 EHR-only processes of care outcomes

7.3.2.1 Summary of baseline data

We will determine the prevalence of patients consulting for a musculoskeletal pain condition and the percentage receiving each of the process of care outcomes (for example, strong opioid prescription) overall, and for each practice. We will stratify by musculoskeletal condition (for example, osteoarthritis, back pain, knee pain), age, and gender.

7.3.2.2 Primary outcomes analysis

To determine each practice's case-mix adjusted percentage of patients receiving a process of care we will develop multivariable generalised logistic fixed effect regression models, with general practice as a fixed effect. We will first determine the association of process of care with the different case-mix factors. Each process of care (for example, strong opioid prescription) will be modelled separately, with the other processes of care included as case-mix variables in the model. We will use a backward stepwise selection process to determine which variables should be included in the final model. Those with a *p*-value of 0.20 or less will be retained. Using this model, the predicted probability of receiving the process of care for each consulting patient can be derived, based on the patient's characteristics and the model beta coefficient estimates. Each general practice's expected percentage receiving the process of care will then be determined by calculating the average predicted probability among all musculoskeletal consulters at that general practice. Comparison to the observed percentage indicates each general practice's absolute (observed - expected) and relative (observed/expected) deviation from the expected percentage receiving the process of care.

We will use funnel plots with 95% control limits to plot the case-mix adjusted deviation from the expected percentage across practices, and identify outlying practices. We will repeat the analyses for each 3- or 6-month interval from July 2016 and for specific musculoskeletal conditions (for example, osteoarthritis, back pain).

8 DATA HANDLING

8.1. Data collection tools and source document identification

Patient-reported data are to be captured through a secure online platform (Keele Health Survey) ensuring that all regulatory requirements are met, including the Data Protection Act 2018, UK General Data Protection Regulation (UKGDPR), NHS Information Governance, and Good Clinical Practice (GCP). Data will also be collected via paper questionnaires; these data will be entered manually into the online platform by a Keele CTU administrator. DOCMAIL is a standards-compliant hybrid mail service, providing document management and ISO 27001 secure mailings.

Patient-reported data will be collected at baseline and monthly for 6 months. Following a retrospective search at the GP practice, eligible patients with an active mobile telephone number registered at the GP practice will be sent a SMS text containing a URL link to an online questionnaire. Patients who do not have an active mobile telephone number at the GP practice will be sent an invitation pack including an invite letter, a Participant Information Sheet, a paper questionnaire, and a pre-paid return envelope. All patients who were sent a SMS text will be sent a reminder text approximately 48 hours after the initial text was sent. If patients do not respond after receiving this text reminder, there will be no further contact with them.

With regard to follow-up, patients will be sent one question at 1, 2, 4, and 5 months. This will either be sent via SMS text or a paper questionnaire. Where there is no response to the monthly question, one reminder SMS text or letter will be sent to the participant. If there is still no response from the participant, then they will continue on the follow-up pathway and will be sent the next month's follow-up questionnaire when it is due. A more substantial follow-up will be done at 3 and 6 months, where patients will be sent either a link via SMS text or email to another online questionnaire or posted a paper questionnaire. Where there is no response to the 3- and 6-month follow-up, two reminder SMS texts or emails or a postcard and then a paper questionnaire will be sent to the participant. If there is

still no response from the participant, at 3 months they will continue on the follow-up pathway, at 6 months there will be no further contact as this is the end of the study.

The consenting process will be clearly outlined, and the potential participant will have to agree to the study and what information is being shared (and with whom) after completing the baseline online questionnaire or at the end of the paper questionnaire, to allow the patient to be 100% clear on exactly what information they are consenting to share.

8.2. Data handling and record keeping

Data management will be carried out in accordance with a Study Data Management Plan, adhering to Keele University Standard Operating Procedures (SOPs).

Study data acquired from participants will be stored on Keele University servers and password protected. Data extracted from GP clinical systems and from NHS Digital databases will be stored on Keele University servers and password protected, and linked for the purposes of analysis, or it will remain within the secure data centre of EMIS X, the electronic health record vendor. The latter approach has only recently been put into effect for urgent COVID research in the OpenSAFELY initiative (OpenSAFELY: Home) but it is believed to attain a higher level of data security and research transparency than previous, conventional approaches to EHR analysis since EHR data remains where it currently resides. Record-level data from NHS Digital will be transferred via secure electronic file transfer. All confidentiality arrangements adhere to relevant data protection regulations and guidelines (Data Protection Act 2018, UK General Data Protection Regulation (UKGDPR), Caldicott, General Medical Council (GMC), Medical Research Council (MRC) UK Policy), Confidentiality NHS Code of Practice, and the Chief Investigator and Study Statistician (Data Custodian) have responsibility to ensure the integrity of the data and that all confidentiality procedures are followed.

All information will be held securely and in strict confidence. Each person who consents to take part in this study will be given a study ID so that data stored from the study will not contain any identifiable information, such as names and addresses. On this basis, these anonymised data will be kept electronically and may be used in other research studies.

The subset of anonymised, non-sensitive data from the locked, validated dataset used to generate the tables, figures, and results for the Final Report to Nuffield Foundation, together with the study protocol, statistical analysis plan, data dictionary, and analysis code, will be made available upon acceptance of the Final Report to the Nuffield Foundation. These datasets will be registered on Keele University's Research Data Repository with a unique Digital Object Identifier (DOI), enhancing its discoverability.

8.3. Access to Data

Keele University is a member of the UK Reproducibility Network and committed to the principles of the UK Concordat on Open Research Data. The School of Medicine and Keele CTU have a longstanding commitment to sharing data from our studies to improve research reproducibility and to maximise benefits for patients, the wider public, and the health and care system.

Metadata, including study protocol, statistical analysis plan, data dictionaries and key study documents (Participant Information Sheet, consent form) will be deposited on a publicly accessible repository if required. De-identified individual participant data (IPD) that underlie the results from this study will be securely stored on servers approved by a government-backed cyber security scheme and made available to bona-fide researchers upon reasonable request via our controlled access procedures. Unless there are exceptional circumstances, data will be available upon publication of

main study findings or within 18 months of study completion (whichever is later) and with no end date. Data requests and enquiries should be directed to medicine.datasharing@keele.ac.uk. We encourage collaboration with those who collected the data, to recognise and credit their contributions.

Any requests for access to the data from anyone outside of the research team (e.g. collaboration, joint publication, data sharing requests from publishers) will follow the Keele University Standard Operating Procedure (SOP) data sharing procedure.

8.4. Data Sharing Agreements

Record-level data requested by the research team will be shared by NHS Digital subject to a Data Sharing Agreement and in the context of a current, valid Data Sharing Framework Contract.

The data generated from this study will remain the responsibility of the Sponsor. Release of data will be subject to a data use agreement between the Sponsor and the third party requesting the data. De-identified individual participant data will be encrypted on transfer.

The full statement on data sharing can be found at https://www.keele.ac.uk/informationgovernance/fortheuniversity/dataprotection/datasharing.

8.5. Archiving

At the end of the study, data will be securely archived in line with the Sponsor's procedures for a minimum of 10 years after end of study declaration and until the sponsor authorises destruction. Archiving will be carried out in accordance with Keele University SOPs.

9 MONITORING & AUDIT

9.1. Study Management

The study will be managed by Keele CTU in accordance with Keele University SOPs. The study Chief Investigator (CI) is responsible for the conduct of the study and will convene a Study Management Group (SMG) comprising members of the research team including the PPIE members. Regular meeting of the SMG will take place throughout the study.

The SMG will oversee the protocol completion, obtaining regulatory approval and site set-up and software development. They will be responsible for the delivery of the study, data collection and the ongoing management. The SMG will monitor recruitment procedures, review against timelines and complete regulatory reporting requirements. In addition, they will also oversee the analyses and the interpretation of the results. The SMG will also ensure there is sufficient staffing support available for the study.

The CRN West Midlands, will co-ordinate the general practice identification process and co-ordinate local implementation and study set-up for the research team.

Our experience demonstrates that this combination of detailed plans with regular SMG meetings ensures successful delivery. Good communication across the study will be facilitated by commonly shared study specific and protected drives on the University's network.

Study monitoring will be carried out in accordance with Study Monitoring and Data Management Plans and Keele University SOPs which lay out the procedures for monitoring the data collection, protocol compliance and data management procedures.

9.2. Independent Advisory Board

In accordance with funder requirements, independent oversight of the programme of research in general and this study in particular will be provided by an Advisory Board comprising senior researchers and practitioners as well as a patient/public representative. The remit of the Advisory Board covers the planning, conduct, and dissemination of the research as laid out in its written Terms of Reference. The Advisory Board will convene initially to provide critical independent feedback on the study protocol and plans. After the initial meeting the Advisory Board will meet annually with the opportunity to schedule meetings at key timepoints in the programme and to agree any additional meetings as deemed necessary by the Chair of the Advisory Board or the Chief Investigator.

9.3. Study timeline

Activity	***Projected Timeline***
**Final draft protocol**	Apr 2021
**Finalise survey instruments and study documentation (PIL, letters, reminders)**	Apr 2021
**Submit to sponsor review**	Apr 2021
**Site recruitment and set-up**	Apr-Sep 2021
**Submit to HRA/Ethics review**	May-Jun 2021
**Keele Health Survey sign-off**	Jun-Jul 2021
**Docmail process set-up**	Jun-Jul 2021
**Management Web Application & Database sign-off**	Jun-Jul 2021
**Retrospective search at first practice**	Jul 2021
**First patient recruited to patient-reported outcomes/experiences subcohort**	Jul-Aug 2021
**Follow-up period can commence**	Aug 2021
**Extracting GP data can commence**	Jul 2021
**Data cleaning process can commence**	Apr 2022
**NHS Digital data requests can commence**	Apr 2022
**Data linkage & Analysis begins**	May 2022
**End of Study**	Mar 2025

10 ETHICAL AND REGULATORY CONSIDERATIONS

Health Research Authority (HRA) approvals will be applied for and obtained before the study commences. HRA Approval is the process for the NHS in England that brings together the assessment of governance and legal compliance, with independent Research Ethics Committee opinion provided through the UK Health Departments' Research Ethics Service.

10.1. Research Ethics Committee (REC) review & reports

This study will be submitted for approval by an appropriate NHS Research Ethics Committee. It will also be submitted for inclusion within the National Institute for Health Research (NIHR) Clinical Research Network (CRN) Portfolio.

- Substantial amendments that require review by REC will not be implemented until the REC grants a favourable opinion for the study (note that amendments may also need to be reviewed by NHS R&D departments before they can be implemented in practice at sites).
- All correspondence with the REC will be retained in the Sponsor Study Master File/local Investigator Site File.
- An annual progress report will be submitted to the REC within 30 days of the anniversary date on which the favourable opinion was given, and annually until the study is declared ended.
- It is the Chief Investigator's responsibility to produce the annual reports as required.
- The Chief Investigator will notify the REC of the end of the study.
- If the study is ended prematurely, the Chief Investigator will notify the REC, including the reasons for the premature termination.
- Within one year after the end of the study, the Chief Investigator will submit a final report with the results, including any publications/abstracts, to the REC.

10.2. Peer review

This study protocol has been subject to internal peer review, external peer review by the funding body (Nuffield Foundation) and peer review by the Advisory Board.

10.3. Public and Patient Involvement

The School of Medicine at Keele University has a strong Patient and Public involvement and Engagement (PPIE) infrastructure, supported by Versus Arthritis Centre of Excellence funding, and which includes a large Research User Group (RUG) advising on all studies within the School. For this study, four patient representatives have been invited from the current RUG members to form a Patient Advisory Group to contribute to the development of certain aspects of the study based on their lived experience of having a chronic painful musculoskeletal condition.

Their key role will include:

- To contribute to discussions on how to maximise inclusion and diversity in this research study
- To contribute to and review participant facing study documents and materials used in the study
- To review the content and order of survey questions
- To provide the patient perspective on the design of the online questionnaire
- To review the recruitment and follow-up methods to be used in the study
- To contribute to and review the dissemination strategy and publications, such as materials or talks with patient forums and practitioners

The Patient Advisory Group has already contributed to the research design by:

- Assessing the proposed research questions in terms of content, layout, style, order of questions, and overall length
- Reviewing the recruitment and follow-up methods proposed for the study including providing advice on promoting and advertising the study to patients
- Discussing issues regarding inclusion and diversity of potential patient groups

The Patient Advisory Group will continue to convene during the study contributing to oversight of the conduct of the study, interpreting findings, and our strategy for dissemination and pathways to achieving impact.

10.4. Regulatory Compliance

Data within the Keele Health Survey is to be captured through secure online forms that meet NHS Information Governance requirements. Patient data (in an electronic format) will be acquired, anonymised, transferred and stored according to the Data Protection Act 2018, UK General Data Protection Regulation (UKGDPR) (Regulation (EU) 2016/679); the Confidentiality NHS Code of Practice; and the Caldicott principles.

Before any site can enrol patients into the study, the CI or designee will apply for HRA approval. For any amendment see section 10.9.

10.5. Protocol compliance

The study will be conducted in compliance with this protocol and GCP guidelines. Deviations from study protocols and GCP occur commonly in health and social care research. The majority of these instances are technical non-compliances that do not result in harm to the study subjects, do not compromise data integrity, or significantly affect the scientific value of the reported results of the study.

Non-compliance may be identified through any study activity but in particular through the use of central monitoring procedures such as consent form review or data management, and self-reporting by the study sites or participants. Deviations from protocols and GCP may occur in research studies. The majority of these instances are technical non-compliances that do not result in harm to the study participants, do not compromise data integrity, or significantly affect the scientific value of the reported results of the study. All deviations will be documented, and appropriate corrective and preventative actions will be taken by Keele CTU with responsibility being taken by the CI.

10.6. Notification of Serious Breaches to GCP and/or the protocol

All instances of protocol deviations will be assessed for severity by the CI (or their delegate), in accordance with the study protocol and using the Sponsor's GCP and Protocol Deviations FOR25.1 Initial Report.

10.7. Data protection and patient confidentiality

See section 8 Data Handling for details of how data is protected and patient confidentiality maintained throughout this study.

All information collected during the course of the study will be kept strictly confidential. Information will be held securely on paper and managed electronically by Keele University through Keele CTU. Keele CTU complies with data protection regulations:

- Appropriate storage, restricted access and disposal arrangements for participant personal and clinical details
- Consent from participants for access to their healthcare records by responsible individuals from the research staff or from regulatory authorities, where it is relevant to study participation
- Consent from participants for the data collected for the study to be used to evaluate safety and develop new research
- All data collection forms that are transferred to and from Keele CTU will be coded with a study number

All research staff/CTU operational staff involved in this study adhere to robust data security procedures and have explicit duties of confidentiality. These practices are written into their employment contracts and are equivalent to the duty placed on NHS staff.

10.8. Indemnity

Keele University has in place Clinical Trials indemnity which provides cover to the University for harm which comes about through the University's, or its staff's, negligence in relation to the design or management of the study and may alternatively, and at the University's discretion provide cover for non-negligent harm to participants.

The NHS has a duty of care to patients treated, whether or not the patient is taking part in a clinical trial, and the NHS organisation (GP practice) remain liable for clinical negligence and other negligent harm to patients under this duty of care.

Agreements between the sponsor and participating NHS organisations detailing study conduct and the responsibilities to be honoured by each party will be fully executed before the study can start at the local NHS Trust.

10.9. Amendments

The need for any potential protocol amendment will be raised with the CI and will be discussed with both the SMG and Sponsor prior to being agreed. Updated versions of the protocol will not be circulated for use until the appropriate regulatory parties have approved the amendment, at which point every effort will be made to implement this updated protocol as soon as is practicably possible, superseding the previous version and documenting the date at which the new protocol was implemented.

10.10. Access to the final dataset

See section 8.4 Data Sharing Agreements.

11 DISSEMINATION POLICY

11.1. Dissemination plan

The School of Medicine, Keele University has a dedicated infrastructure, linked to strong regional, national and international health care and academic networks, which facilitate dissemination of our research findings to key policy, commissioning clinical, health education and patient stakeholders. The research team will be able to access our dedicated infrastructure to identify and promote research outputs that lend themselves to translation by health providers.

Expected main outcomes from this study include:

1. New data, information, and intelligence on inequalities and variations in musculoskeletal health outcomes, experiences and care
2. New insights into the feasibility, validity, and persuasiveness of new musculoskeletal health indicators and data visualisations

The key audiences for our research are:

a) patients with musculoskeletal conditions and the wider public;
b) healthcare professionals, with particular emphasis on general practitioners and first contact practitioners;
c) local health policymakers, including clinical commissioners and PCN leads;
d) external statutory bodies (e.g. NHS England, Public Health England), patient groups (e.g. ARMA) and charities (e.g. Versus Arthritis);
e) Academia

Planned outputs:

- Written aggregate-level reports and data visualisations to participating GP practices and PCNs (a,b)
- Press releases, briefings, articles, and interviews for local radio and newspapers (a,b,c)
- Written and oral presentation to local policy/planning meetings
- Use of electronic media including a study website, institutional websites, social media including Twitter, YouTube video (all)
- Links with key local, national and international organisations including the Versus Arthritis National MSK Health Data Group, West Midlands Academic Health Science Network, Applied Research Collaboration, Keele Deal: Health, Public Health England, NICE, to contribute to and capitalise on their networks (all)
- Publications including full report, executive summary and plain English summary, peer-reviewed journals, and local NHS and research newsletters (all)
- Presentations at high-profile scientific and health policy conferences: NHS Evidence, Society for Academic Primary Care, Chartered Society of Physiotherapy, Public Health England (b,c,d,e)

11.2. Authorship eligibility guidelines and any intended use of professional writers

Authorship will be available to those who fulfil the International Committee of Medical Journal Editors (ICMJE) criteria. No-one who fulfils the ICMJE criteria should be excluded from authorship credit and, of equal importance, no-one who fails to fulfil the four criteria should receive authorship credit. This includes academic staff and students as well as CTU, administrative, informatics, IT and nursing staff, and patient/public representatives where they fulfil all four criteria above. However, individuals have the right to choose not to be an author on a particular paper.

Staff heavily involved in the practicalities of study operationalisation and delivery, including dedicated study co-ordinators, will be considered for co-authorship of protocol papers on the condition they can contribute to critical revision of drafts, approve the final version, and be accountable for the content.

There is no intention to use professional writers.

12 REFERENCES

1. Blyth FM, Briggs AM, Schneider CH, Hoy DG, March LM. The Global Burden of Musculoskeletal Pain-Where to From Here? Am J Public Health. 2019 Jan;109(1):35-40.

2. Institute for Health Metrics and Evaluation (IHME). GBD Compare Data Visualization. Seattle, WA: IHME, University of Washington, 2020. Available from http://vizhub.healthdata.org/gbd-compare. Last accessed: 2 Feb 2021.
3. Health & Safety Executive. LFS - Labour Force Survey - Self-reported work-related ill health and workplace injuries: Index of LFS tables. Updated 11/20. Available at LFS - Labour Force Survey - Self-reported work-related ill health and workplace injuries: Index of LFS tables (hse.gov.uk). Last accessed: 2 Feb 2021.
4. NHS England. 2013-14 CCG Programme Budgeting Benchmarking Tool, 2015. Available at: https://www.england.nhs.uk/prog-budgeting/. Last accessed: 2 Feb 2021.
5. Arthritis Research UK National Primary Care Centre. Musculoskeletal Matters. Bulletin 1: What do general practitioners see? Oct 2009. Available at: https://www.keele.ac.uk/media/keeleuniversity/ri/primarycare/MusculoskeletalMatters_Issue1_Final.pdf. Last accessed: 2 Feb 2021.
6. Jordan K. Jordan KP, Kadam UT, Hayward R, Porcheret M, Young C, Croft P. Annual consultation prevalence of regional musculoskeletal problems in primary care: an observational study. BMC Musculoskelet Disord. 2010 Jul 2;11:144.
7. Chief Medical Officer Annual report: Volume One, 2011 ‘On the state of the public’s health’, Nov 2012.
8. Newton JN, Briggs AD, Murray CJ, et al. Changes in health in England, with analysis by English regions and areas of deprivation, 1990-2013: a systematic analysis for the Global Burden of Disease Study 2013. Lancet. 2015 Dec 5;386(10010):2257-74.
9. Steel N, Ford JA, Newton JN, et al. Changes in health in the countries of the UK and 150 English Local Authority areas 1990-2016: a systematic analysis for the Global Burden of Disease Study 2016. Lancet. 2018 Nov 3;392(10158):1647-1661.
10. Musculoskeletal Health: A 5 year strategic framework for prevention across the lifecourse. Available at: https://assets.publishing.service.gov.uk/government/uploads/system/uploads/attachment_data/file/810348/Musculoskeletal_Health_5_year_strategy.pdf. Last accessed: 2 Feb 2021.
11. Blumenberg, C., Barros, A.J.D. Response rate differences between web and alternative data collection methods for public health research: a systematic review of the literature. *Int J Public Health* 63, 765–773 (2018). https://doi.org/10.1007/s00038-018-1108-4
12. Office for National Statistics. INTERNET ACCESS - HOUSEHOLDS AND INDIVIDUALS, 2020: PUBLISHED 7th AUGUST 2020. Available at: Internet access - households and individuals - Office for National Statistics (ons.gov.uk) Last accessed: 11 Feb 2021.
13. NHS Digital. What we mean by digital inclusion. Available at: What we mean by digital inclusion - NHS Digital Last accessed: 11 Feb 2021.
14. National Institute for Health Research (2020) Improving inclusion of under-served groups in clinical research: Guidance from the NIHR INCLUDE project. UK: National Institute for Health Research. Available at: www.nihr.ac.uk/documents/improving-inclusion-of-under-served-groups-in-clinical-research-guidance-from-include-project/25435 Last accessed: 21 Feb 2021.
15. TrialForge. The INCLUDE Ethnicity framework. Available at: The INCLUDE Ethnicity Framework - Trial Forge Last accessed: 21 Feb 2021.

16. Nicolaas, G, Calderwood L, Lynn P, Roberts C. Web surveys for the general population: How, why, and when? London: National Centre for Research Methods Report, 2014.
17. Lynn, P. (2020). Evaluating push-to-web methodology for mixed-mode surveys using address-based samples. *Survey Research Methods*, *14*(1), 19-30. https://doi.org/10.18148/srm/2020.v14i1.7591
18. Edwards PJ, Roberts I, Clarke MJ, Diguiseppi C, Wentz R, Kwan I, Cooper R, Felix LM, Pratap S. Methods to increase response to postal and electronic questionnaires. Cochrane Database Syst Rev. 2009 Jul 8;(3):MR000008. doi: 10.1002/14651858.MR000008.pub4.
19. Krebs EE, Lorenz KA, Bair MJ, Damush TM, Wu J, Sutherland JM, Asch SM, Kroenke K. Development and initial validation of the PEG, a three-item scale assessing pain intensity and interference. J Gen Intern Med. 2009 Jun;24(6):733-8. doi: 10.1007/s11606-009-0981-1.
20. Von Korff M, DeBar LL, Krebs EE, Kerns RD, Deyo RA, Keefe FJ. Graded chronic pain scale revised: mild, bothersome, and high-impact chronic pain. Pain. 2020 Mar;161(3):651-661. doi: 10.1097/j.pain.0000000000001758.
21. Hill JC, Kang S, Benedetto E, Myers H, Blackburn S, Smith S, Dunn KM, Hay E, Rees J, Beard D, Glyn-Jones S, Barker K, Ellis B, Fitzpatrick R, Price A. Development and initial cohort validation of the Arthritis Research UK Musculoskeletal Health Questionnaire (MSK-HQ) for use across musculoskeletal care pathways. BMJ Open 2016;6(8):e012331.
22. NHS. GP Patient Survey. Available at: GP Patient Survey (gp-patient.co.uk) Last accessed:25 Mar 2021
23. Burt J, Lloyd C, Campbell J, Roland M, Abel G. Variations in GP-patient communication by ethnicity, age, and gender: evidence from a national primary care patient survey. *Br J Gen Pract*. 2016;66(642):e47-e52. doi:10.3399/bjgp15X687637
24. Morris NS, MacLean CD, Chew LD, Littenberg B. The single item literacy screener: evaluation of a brief instrument to identify limited reading ability. BMC Fam Pract. 2006;7:21
25. Fletcher R. Ethnicity harmonised standard. Government Statistical Service, London, Apr 2019. Available at: Ethnicity harmonised standard – GSS (civilservice.gov.uk) Last accessed: 25 Mar 2021.
26. Thomas R, 1999. Income-commentary. Question Bank, University of Surrey.
27. Reilly MC, Zbrozek AS, Dukes EM. The validity and reproducibility of a work productivity and activity impairment instrument. PharmacoEconomics 1993; 4(5):353-65.
28. Versus Arthritis. Recommended Musculoskeletal Indicator Set. 2018. Available at: MSK Recommended Indicator Set (versusarthritis.org) Last accessed: 24 March 2021.
29. Oliver S, Kavanagh J, Caird J, Lorenc T, Oliver K, Harden A, et al. Health promotion, inequalities and young people's health. A systematic review of research. London: University of London; 2008.
30. O'Neill J, Tabish H, Welch V, Petticrew M, Pottie K, Clarke M, et al. Applying an equity lens to interventions: using PROGRESS ensures consideration of socially stratifying factors to illuminate inequities in health. J Clin Epidemiol. 2014 Jan;67(1):56-64.
31. Public Health England. *Return on Investment of intervention for the prevention and treatment of musculoskeletal conditions: final report.* Oct 2017.
32. NIHR. *Moving Forward. Physiotherapy for Musculoskeletal Health and Wellbeing.* July 2018.

33. Jordan KP, Jöud A, Bergknut C, Croft P, Edwards JJ, Peat G, Petersson IF, Turkiewicz A, Wilkie R, Englund M. International comparisons of the prevalence of health care for musculoskeletal disorders using population-based health care data from England and Sweden, Annals of the Rheumatic Diseases, 2014;73:1:212-218.
34. Vickers AJ. How many repeated measures in repeated measures designs? Statistical issues for comparative trials. BMC Med Res Methodol. 2003 Oct 27;3:22. doi: 10.1186/1471-2288-3-22.
35. Neuburger J, Cromwell DA, Hutchings A, et al. Funnel plots for comparing provider performance based on patient-reported outcome measures BMJ Quality & Safety 2011;20:1020-1026.
