# Supplementary material for: Socioeconomic inequalities in outcomes, experiences and treatment among adults consulting primary care for a musculoskeletal pain condition: a prospective cohort study
Source: BMJ Open. 2025 Jul 15;15(7):e095132. doi: 10.1136/bmjopen-2024-095132 (PMC12265837; doi:10.1136/bmjopen-2024-095132)
Supplement: online supplemental file 5 [file bmjopen-15-7-s005.docx]

**Socioeconomic inequalities in outcomes, experiences, and treatment among adults consulting primary care for a musculoskeletal pain condition: a prospective cohort study**

George M Peat, Jonathan C Hill, Dahai Yu, Simon Wathall, Emma Parry, James Bailey, Clare Thompson, Kelvin P Jordan, and the MIDAS Patient Advisory Group

**Supplementary Appendix 5: Additional analyses**

| **Table S1:** Baseline characteristics of (a) all eligible survey respondents (n=2008), (b) all eligible survey respondents consenting to electronic health record linkage (n=1875), (c) all eligible survey respondents consenting to electronic health record linkage and responding at three and six-month follow-up (n=945) |
| --- |
| **Table S2:** Association of deprivation with MSK-HQ score and interaction with wave: estimates from imputed analysis |
| **Table S3:** Association of deprivation with dissatisfaction with consultation and with opioid prescription within 14 days of index consultation: estimates from imputed analyses |

**Table S1:** Baseline characteristics of (a) all eligible survey respondents (n=2008), (b) all eligible survey respondents consenting to electronic health record linkage (n=1875), (c) all eligible survey respondents consenting to electronic health record linkage and responding at three and six-month follow-up (n=945)

|  | Baseline responders | Baseline responders + EHR linkage | Responded all 3 waves + EHR linkage |
| --- | --- | --- | --- |
| *n* | 2008 | 1875 | 945 |
| Age, Mean (SD) | 57.7 (15.5) | 57.7 (15.5) | 61.1 (13.8) |
| Female | 1322 (66) | 1233 (66) | 628 (66) |
| Deprivation^a^ IMDq1 (Most)  IMDq2  IMDq3  IMDq4  IMDq5 (Least) | 562 (29)  388 (20)  419 (21)  343 (17)  249 (13) | 530 (28)  383 (20)  398 (21)  320 (17)  244 (13) | 227 (24)  187 (20)  211 (22)  184 (20)  136(14) |
| BMI, Mean (SD) | 29.2 (7.0) | 29.2 (6.9) | 29.4 (6.8) |
| Ethnicity, White | 1919 (96) | 1788 (95) | 921 (97) |
| Time between consultation & survey (days), Mean (SD) | 9.6 (14.3) | 9.4 (11.1) | 10.2 (13.4) |
| Paper survey | 301 (15) | 282 (15) | 161 (17) |
| Previous MSK surgery | 252 (13) | 238 (13) | 137 (14) |
| Duration of symptoms  <3 months  4-6 months  7-12 months  13 months-3 years  Over 3 years | 810 (40)  216 (11)  262 (13)  316 (16)  404 (20) | 758 (40)  205 (11)  251 (13)  290 (15)  371 (20) | 354 (37)  97 (10)  140 (15)  150 (16)  204 (22) |
| Previous episodes 0  1  2-3  4-9  10+ | 369 (18)  136 (7)  322 (16)  255 (13)  926 (46) | 345 (18)  129 (7)  306 (16)  233 (12)  862 (46) | 159 (17)  60 (6)  150 (16)  124 (13)  452 (48) |
| Pain Sites 1 site  2 sites  >2 sites | 1006 (50)  408 (20)  594 (30) | 936 (50)  390 (21)  549 (29) | 471 (50)  194 (21)  280 (30) |
| Physical activity (days) Mean (SD) | 2.2 (2.4) | 2.2 (2.4) | 2.2 (2.4) |
| Comorbidity Count 0  1  2  ≥3 | N/A | 829 (44)  577 (31)  329 (18)  140 (7) | 415 (44)  282 (30)  177 (19)  71 (8) |
| DPC FTE per 1,000 registered population, Mean (SD) | 21.7 (13.4) | 21.8 (13.7) | 22.2 (13.3) |
| GP FTE per 1,000 registered population, Mean (SD) | 61.3 (27.2) | 60.4 (27.5) | 62.2 (26.9) |
| Practice-level MSK consultation per 10,000, Mean (SD) | 2693 (843) | 2683 (845) | 2701 (801) |
| Baseline MSK-HQ, Mean (SD) | 25.3 (10.8) | 25.8 (10.6) | 26.2 (10.6) |
| Month-3 MSK-HQ, Mean (SD) | 29.9 (12.7) | 30.2 (12.7) | 30.5 (12.6) |
| Month-6 MSK-HQ, Mean (SD) | 31.1 (13.3) | 31.4 (13.3) | 31.9 (13.4) |
| Dissatisfaction with consultation | 126 (6) | 119 (6) | 44 (5) |
| Opioid analgesic | N/A | 493 (26) | 243 (26) |
| Numbers are n (%) unless otherwise stated  *^a^* Index of Multiple Deprivation (IMD) available for *n*=1961  **BMI** Body Mass Index; **DPC** Direct Patient Care; **EHR** Electronic health record; **FTE** Full Time Equivalent; **GP** General Practitioner; **IMDq1** Index of Multiple Deprivation, grouped by quintile value, IMDq1=most deprived; **MSK** Musculoskeletal; **MSK-HQ** Musculoskeletal Health Questionnaire; **SD** Standard Deviation | | | |

**Table S2:** Association of deprivation with MSK-HQ score and interaction with wave: estimates from imputed analysis

|  | | Model | | | | | | | |
| --- | --- | --- | --- | --- | --- | --- | --- | --- | --- |
|  | | *β*^a^ (95% CI) | | *β*^b^ (95% CI) | | *β^c^* (95% CI) | | *β^d^* (95% CI) | |
| Deprivation group | IMDq1 (Most) | Ref | Ref | | Ref | | Ref | |  |
|  | IMDq2 | 2.72 (1.08, 4.36) | | 2.35 (0.72, 3.98) | | 1.17 (-0.23, 2.58) | | 1.11 (-0.30, 2.52) | |
| IMDq3 | | 3.60 (2.00, 5.20) | | 2.95 (1.34, 4.55) | | 0.80 (-0.59, 2.19) | | 0.77 (-0.62, 2.16) | |
| IMDq4 | | 4.50 (2.78, 6.23) | | 3.66 (1.93, 5.40) | | 2.06 (0.56, 3.55) | | 2.00 (0.51, 3.50) | |
| IMDq5 (Least) | | 4.67 (2.78, 6.57) | | 3.99 (2.10, 5.89) | | 1.30 (-0.35, 2.95) | | 1.21 (-0.45, 2.87) | |
|  | |  | |  | |  | |  | |
| Wave Baseline  3-month  6-month | | Ref  3.94 (2.41, 5.46)  4.58 (3.05, 6.11) | | Ref  3.94 (2.42, 5.45)  4.58 (3.05, 6.10) | | Ref  3.99 (2.65, 5.33)  4.62 (3.17, 6.07) | | Ref  3.98 (2.65, 5.32)  4.62 (3.16, 6.08) | |
|  | |  | |  | |  | |  | |
| Wave * Deprivation 3-month_IMDq2  3-month_IMDq3  3-month_IMDq4  3-month_IMDq5  6-month_IMDq2  6-month_IMDq3  6-month_IMDq4  6-month_IMDq5 | | -0.56 (-2.92, 1.80)  1.23 (-1.02, 3.48)  -0.15 (-2.57, 2.27)  1.52 (-1.15, 4.19)  0.40 (-1.93, 2.74)  2.05 (-0.25, 4.36)  0.71 (-1.76, 3.17)  2.43 (-0.26, 5.12) | | -0.56 (-2.91, 1.79)  1.23 (-1.00, 3.47)  -0.15 (-2.56, 2.26)  1.52 (-1.14, 4.18)  0.40 (-1.92, 2.72)  2.05 (-0.24, 4.34)  0.71 (-1.75, 3.16)  2.43 (-0.24, 5.11) | | -0.70 (-2.72, 1.33)  1.24 (-0.78, 3.26)  -0.11 (-2.22, 2.00)  1.53 (-0.82, 3.89)  0.29 (-1.79, 2.37)  1.99 (-0.13, 4.11)  0.63 (-1.61, 2.87)  2.44 (0.01, 4.86) | | -0.69 (-2.71, 1.33)  1.24 (-0.78, 3.27)  -0.10 (-2.21, 2.01)  1.55 (-0.80, 3.90)  0.29 (-1.79, 2.38)  1.99 (-0.14, 4.11)  0.63 (-1.61, 2.88)  2.44 (0.01, 4.87) | |
|  | |  | |  | |  | |  | |
| Variance partition (practice level) | | 0.01 | | 0.01 | | 0.01 | | 0.01 | |
| **CI** Confidence Interval; **IMDq1** Index of Multiple Deprivation grouped by quintile value, IMDq1=most deprived; **OR** Odds Ratio  ^a^ unadjusted, ^b^ adjusted for sociodemographic and survey-related covariates, ^c^ further adjusted for clinical case-mix covariates, ^d^ further adjusted for practice-level covariates | | | | | | | | | |

**Table S3:** Association of deprivation with dissatisfaction with consultation and with opioid prescription within 14 days of index consultation: estimates from imputed analyses

|  | OR^a^ (95% CI) | OR^b^ (95% CI) | OR*^c^* (95% CI) | OR*^d^* (95% CI) |
| --- | --- | --- | --- | --- |
| **Dissatisfaction with consultation** |  |  |  |  |
| Deprivation group IMDq1 (Most)  IMDq2  IMDq3  IMDq4  IMDq5 (Least) | Ref  0.68 (0.40, 1.14)  0.69 (0.42, 1.15)  0.43 (0.23, 0.82)  0.64 (0.34, 1.19) | Ref  0.84 (0.49, 1.42)  0.94 (0.56, 1.59)  0.60 (0.32, 1.15)  0.89 (0.47, 1.68) | Ref  0.87 (0.50, 1.49)  0.99 (0.58, 1.69)  0.62 (0.32, 1.20)  0.97 (0.50, 1.87) | Ref  0.88 (0.51, 1.53)  1.01 (0.59, 1.73)  0.63 (0.32, 1.22)  1.04 (0.53, 2.02) |
| Variance partition (practice level) | 0.01 | 0.003 | 0.01 | 0.001 |
|  |  |  |  |  |
| **Opioid prescription** |  |  |  |  |
| Deprivation group IMDq1 (Most)  IMDq2  IMDq3  IMDq4  IMDq5 (Least) | Ref  0.86 (0.64, 1.18)  1.00 (0.74, 1.34)  0.78 (0.56, 1.09)  0.58 (0.40, 0.86) | Ref  0.84 (0.61, 1.14)  0.94 (0.69, 1.28)  0.74 (0.52, 1.04)  0.55 (0.37, 0.81) | Ref  0.95 (0.68, 1.31)  1.14 (0.83, 1.57)  0.84 (0.59, 1.21)  0.72 (0.48, 1.09) | Ref  0.94 (0.68, 1.30)  1.15 (0.84, 1.58)  0.85 (0.59, 1.21)  0.74 (0.49, 1.12) |
| Variance partition (practice level) | 0.01 | 0.01 | 0.01 | 0.01 |
| **CI** Confidence Interval; **IMDq1** Index of Multiple Deprivation grouped by quintile value, IMDq1=most deprived; **OR** Odds Ratio  ^a^ unadjusted, ^b^ adjusted for sociodemographic and survey-related covariates, ^c^ further adjusted for clinical case-mix covariates, ^d^ further adjusted for practice-level covariates | | | | |
