# Supplementary material for: Socioeconomic inequalities in outcomes, experiences and treatment among adults consulting primary care for a musculoskeletal pain condition: a prospective cohort study
Source: BMJ Open. 2025 Jul 15;15(7):e095132. doi: 10.1136/bmjopen-2024-095132 (PMC12265837; doi:10.1136/bmjopen-2024-095132)
Supplement: online supplemental file 7 [file bmjopen-15-7-s007.docx]

**GRIPP2 2017 (SF) Checklist**

| Section and topic | Item | Reported on page no. |
| --- | --- | --- |
| 1: Aim | Report the aim of PPI in the study | 9 |
| 2: Methods | Provide a clear description of the methods used for PPI in the study | 9 |
| 3: Study results | Outcomes—Report the results of PPI in the study, including both positive and negative outcomes | 11,12 |
| 4: Discussion and conclusions | Outcomes—Comment on the extent to which PPI influenced the study overall. Describe positive and negative effects | 3,11,12 |
| 5: Reflections/critical perspective | Comment critically on the study, reflecting on the things that went well and those that did not, so others can learn from this experience | 11,12 |

BMJ 2017;358:j3453 http://dx.doi.org/10.1136/bmj.j3453
