# Supplementary material for: Socioeconomic inequalities in outcomes, experiences and treatment among adults consulting primary care for a musculoskeletal pain condition: a prospective cohort study
Source: BMJ Open. 2025 Jul 15;15(7):e095132. doi: 10.1136/bmjopen-2024-095132 (PMC12265837; doi:10.1136/bmjopen-2024-095132)
Supplement: Supplementary file 8 [file bmjopen-2024-095132.reviewer_comments.pdf]

PEER REVIEW HISTORY

BMJ Open publishes all reviews undertaken for accepted manuscripts. Reviewers are asked to complete a checklist review form and are provided with free text boxes to elaborate on their assessment. These free text comments are reproduced below.

ARTICLE DETAILS

Title (Provisional)

Socioeconomic inequalities in outcomes, experiences, and treatment among adults consulting primary care for a musculoskeletal pain condition: a prospective cohort study

Authors

Peat, George; Hill, Jonathan C; Yu, Dahai; Wathall, Simon; Parry, Emma; Bailey, James; Thompson, Clare; Jordan, Kelvin P

VERSION 1 - REVIEW

**Reviewer**	**1**
**Name**	**Sleeswijk Visser, Tjerk**
**Affiliation**	**Erasmus MC**
**Date**	**23-Jan-2025**
**COI**	**None**

Comments to the authors:

I would like to congratulate the authors on this interesting and well-constructed study. Generally the manuscript is well-written, clear and of added benefit to the field. Please see my specific comments/thoughts below.

Abstract

Line 36: If not getting in trouble with the word count for the abstract, perhaps include the MSK-HQ scores at baseline

Line 43: suggest to rewrite: ‘Only 6% of all patient reported…’

Introduction

Throughout the introduction the terms ‘affluent’, ’deprived’ and ‘disadvantaged’ are used. Try to be consistent with terminology to make it more clear for readers who are not familiair with these different terms

Line 12: remove space before citation

Line 12-16: This is quite a long sentence and hard to follow. Suggest to break down in two seperate ones.

Line 27: ‘to have regard to the need to reduce..’ This is hard to follow

Page 6, Line 10: Write GPs fullout the first time you use the abbreviation

Methods

Page 7, Line 36: Is this dichotomisation based on previous research? Is there a rationale to include ’neither good nor poor’ into the ‘satisfied’ group?

Page 7, Line 55. You could use references of previous studies who used this approach to support your use of this method. (e.g. Carr-Hill R . The measurement of inequities in health: lessons from the British experience. Soc Sci Med 1990;31:393–404.)

Page 8, Line 36. This is a very long sentence.

Page 9, Line 43: Did you investigate if data was missing completely at random? If yes, how did you do this? Can you make strong statements if you expect a lot of missing data or should results be interpreted as hypothesis-generating rather than definitive (Jakobsen JC , Gluud C , Wetterslev J , et al . When and how should multiple imputation be used for handling missing data in randomised clinical trials - a practical guide with Flowcharts. BMC Med Res Methodol 2017;17:162)

Results

Page 10, Line 52: ‘were also more likely to have longstanding pain, more previous pain episodes, multiple site pain, and comorbidities.’ Is this statistically significant? This statement is not entirely reflected by the percentages in Table 1. More likely is also a bit of a vague term. Perhaps there is a small trend towards these differences.

Discussion

Page 12, Line 33: ‘… and even widened’; Can you state this based on the stastically insignificant results? Perhaps rephrase a bit, eg. showed a trend towards…

Page 13, Line 10. Was there a difference between pen-and-paper completed surveys and digital ones between the quintiles?

Page 13, Line 15:’ Selective loss to follow-up appeared a lesser threat to validity’ In table 1 it appears as at 6-months there is a 14% difference in response rate in favour of the least deprived group (compared to the most deprived)?

General remark: Is the difference in the MSK-HQ-score clinically relevant? Based on previous research the minimal clinically inmportant difference is 5.5 (which is larger than the difference found in this study)? Does this influence the results or the conclusions you can make based on the data in this study?

Also in you study protocol you stated (under primary outcome measures) the aim to determine the percentage of patients who achieve the minimal important change. I can not find anything on this in the manuscript?

**Reviewer**	**2**
**Name**	**Brummet, Chad**
**Affiliation**	**University of Michigan Michigan Medicine**
**Date**	**25-Mar-2025**
**COI**	**consultant for Vertex pharmaceuticals and Merck Pharmaceuticals. expert medicolegal testimony**

Thank you for submitting this manuscript to BMJ Open. The topic is of high importance, as socioeconomic disparities in pain remain understudied. There are a number of strengths of this manuscript, including the prospective design, large cohort and multi-center approach. The baseline discrepancy is important but not surprising, but the widened gap over 6 months is really of interest.

Overall comments and major critiques:

1. Opioid prescription as an indicator of low-value care- this is interesting. I do a great deal of work opioid stewardship and am a chronic pain physician. That said, there is a lot of nuance to the rationale for an opioid prescription, including previous therapies tried, comorbidities, etc. While differential prescriptions by socioeconomic status is interesting, I'm not sure I love terming it low value care.

2. Responder bias- while prospective data are challenging a 50% loss to follow up creates meaningful responder bias.

3. Non-opioid treatments- I appreciate the focus on opioids; however, it would be far more interesting to categorize the other types of treatments given in the first 14 days to better understand what options other patients not receiving opioids are being given and whether this varies by SES. Large categories would be ok---non-opioid analgesics, behavioral therapies, physical therapy, other??

4. Satisfaction with care while commonly measured is not really a good measure of the quality of care or action patient satisfaction. There are too many variables at play to come out through simple, semi-structured approaches.

5. Abstract- minor point, but would focus on the primary outcomes and provide detail. Secondary outcomes can move to the body of the manuscript.

Additional comments:

1. Methods- I see ethnicity but not race. May be differences in the way you handle demographics.

VERSION 1 - AUTHOR RESPONSE

Reviewer: 1

Abstract

1. Line 36: If not getting in trouble with the word count for the abstract, perhaps include the MSK-HQ scores at baseline
   Response: We have reduced information presented on secondary outcomes (as per Reviewer 2, #5) and added the mean (SD) MSK-HQ scores for the most and least deprived patients
   Revision: "Compared to patients from the least deprived neighbourhoods, patients from the most deprived neighbourhoods had significantly poorer MSK-HQ scores at baseline *(mean 22.6 (SD 10.4) vs 27.6 (10.1))*."

2. Line 43: suggest to rewrite: 'Only 6% of all patient reported…'
   Response: This has been removed as per Reviewer 2, #5.

   Introduction
3. Throughout the introduction the terms 'affluent', 'deprived' and 'disadvantaged' are used. Try to be consistent with terminology to make it more clear for readers who are not familiar with these different terms
   Response: we have revised this to consistently used 'deprived'
   Revision: "Musculoskeletal pain conditions – low back pain, neck pain, osteoarthritis and other regional pain conditions - are a major cause of population disability(1) and typically occur more frequently, and have a greater impact, in socioeconomically *deprived* individuals and communities.(2) Evidence from previous studies in the UK suggests that healthcare may follow a Disproportionate Care Law(3) in which people from *more deprived backgrounds* may have higher healthcare use but the care is of lower quality and insufficient for their additional complexity and less favourable prognosis at the time of presenting to services. For example, compared to the *least deprived* areas, people living in the most deprived neighbourhoods in England have consistently higher primary care consultation rates for musculoskeletal pain conditions.(4)"

4. Line 12: remove space before citation
   Revision: revised as per suggestion

5. Line 12-16: This is quite a long sentence and hard to follow. Suggest to break down in two separate ones.
   Response: revised as per suggestion

Revision: "For example, compared to the most affluent areas, people living in the most deprived neighbourhoods in England have consistently higher primary care consultation rates for musculoskeletal pain conditions.(4) *They also* present with higher levels of severity, complexity, and comorbidity,(5) *and they* are more likely to be treated with long-term opioid analgesia.(6–9)"

6. Line 27: 'to have regard to the need to reduce..' This is hard to follow
Response: We agree, although it is the form of words used in the Act of Parliament. We have revised this sentence to put it into plainer language.
Revision: "The UK Health and Social Care Act 2022 introduced a legal duty on the NHS to *consider how* to reduce health inequalities"

7. Page 6, Line 10: Write GPs fullout the first time you use the abbreviation
Response: revised as suggested

Methods

8. Page 7, Line 36: Is this dichotomisation based on previous research? Is there a rationale to include 'neither good nor poor' into the 'satisfied' group?
Response: Our focus was on expressed dissatisfaction with care rather than satisfaction or indifference. We recognise though that we have used varying language throughout the manuscript (e.g. 'poor experience', 'experience', 'satisfaction') so have revised this to be consistent throughout

9. Page 7, Line 55. You could use references of previous studies who used this approach to support your use of this method. (e.g. Carr-Hill R . The measurement of inequities in health: lessons from the British experience. Soc Sci Med 1990;31:393–404.)
Response: Thank you for this suggestion. We have added the Carr-Hill reference which supports the 'quintile solution' as a means of comparing morbidity inequalities. In the Discussion we have also added a citation of Ingleby et al. (2020) who provide empirical evidence on the limitations of area-level deprivation which we noted was a potential limitation of this approach.
Revision: "Participants were categorised into 5 quintile groups from most to least deprived based on the national ranking of neighbourhoods*(16)*."
"However, we acknowledge that an individual-level measure of socioeconomic position (e.g. educational attainment, occupational class) could be more sensitive*(24)*; something our PAG felt was important."

10. Page 8, Line 36. This is a very long sentence.
Response: we have tried to break this down to make it more digestible
Revision: "*We based our sample size on the following assumptions*: *the* median registered population size for general practices in North Staffordshire & Stoke-on-Trent *is 7300*; 80% *of registered patients* are aged 18 and over; the annual MSK consultation prevalence is 15%;(21) a 25% participation rate; *an* 80% consent *rate* to EHR linkage; 50% follow-up at 6 months; each of 26 GP practices would recruit for a 3 month period. *Based on these*

estimates and assumptions we anticipated 1424 participants at baseline, 1139 consenting to EHR linkage, and 569 responding at 6-month follow-up."

11. Page 9, Line 43: Did you investigate if data was missing completely at random? If yes, how did you do this? Can you make strong statements if you expect a lot of missing data or should results be interpreted as hypothesis-generating rather than definitive (Jakobsen JC , Gluud C , Wetterslev J , et al . When and how should multiple imputation be used for handling missing data in randomised clinical trials - a practical guide with Flowcharts. BMC Med Res Methodol 2017;17:162)
Response: The main data missing were the dependent outcome variables (MSK-HQ at 3 and 6 months) due to loss to follow-up. Description of baseline characteristics for complete cases and total eligible respondents (Supplementary Appendix 5, Table S1) suggested that the data could not be assumed to be MCAR. We therefore used multiple imputation using chained equations based on the assumption of MAR. While the assumption of MAR is unverifiable, the inclusion of a large number of predictors (including case-mix adjustment factors that have been demonstrated to be strong predictors of outcome) in our imputation model is likely to strengthen belief in the assumption of MAR. However, we do accept that even though complete case analysis and multiple imputation findings were consistent, the size of missing outcome data should urge caution. The idea that such findings should be considered hypothesis-generating is attributed by Jakobsen et al (2017) to Clark & Altman (2003) (we could find no such statement in Clark & Altman's article) but we are happy to state this.
Revision: Added to the methods: "The primary analyses used the observed data from baseline responders with linkage to EHR. We described baseline characteristics among complete cases and total eligible respondents to judge whether the complete case dataset could reasonably be judged as representative of the total eligible respondent dataset (i.e. data missing completely at random). In sensitivity analysis we assumed data were missing at random and used multiple imputation to impute information…"
Added to the discussion: "Given high levels of missing data for outcomes, our findings should be treated as hypothesis-generating rather than definitive.(23)"

Results

12. Page 10, Line 52: 'were also more likely to have longstanding pain, more previous pain episodes, multiple site pain, and comorbidities.' Is this statistically significant? This statement is not entirely reflected by the percentages in Table 1. More likely is also a bit of a vague term. Perhaps there is a small trend towards these differences.
Response: these are descriptive comparisons of the sample characteristics and not intended for inferential testing and we would not seek to calculate confidence intervals or p values. The differences in some cases may be small (and in the case of comorbidity count 3+ is equal to the second most deprived group) but it is a fact that the participants from the most deprived neighbourhoods had the highest proportion reporting symptoms lasting >3 years, had the highest proportion reporting multiple previous episode of pain, had the highest proportion reporting multiple pain sites, and had the highest proportion reporting 1 or more comorbidity. We could use this phrasing

instead to remove ambiguity but we worry it might feel overly pedantic and the sense in both forms is the same.
Revision: we respectfully request to keep the wording as it is

Discussion

13. Page 12, Line 33: '… and even widened'; Can you state this based on the statistically insignificant results? Perhaps rephrase a bit, eg. showed a trend towards…
    Response: we agree, given the 95% confidence intervals around these estimates include zero we should make this clearer. We have revised this accordingly.
    Revision: "The inequality gap in musculoskeletal health did not *decrease* over the following six months after consultation, but showed *a small statistically non-significant increase*, a finding which persisted after case-mix adjustment."

14. Page 13, Line 10. Was there a difference between pen-and-paper completed surveys and digital ones between the quintiles?
    Response: Figures presented table 1 suggest that paper completion accounted for a slightly lower proportion of respondents in the most deprived group compared with among the least deprived group.

	Total	IMDq1 (Most)	IMDq2	IMDq3	IMDq4	IMDq5 (Least)
Paper survey	282 (15)	55 (10)	54 (14)	72 (18)	56 (18)	45 (18)

    Revision: No revision.

15. Page 13, Line 15:' Selective loss to follow-up appeared a lesser threat to validity' In table 1 it appears as at 6-months there is a 14% difference in response rate in favour of the least deprived group (compared to the most deprived)?
    Response: We intended this more relative to the scale of non-participation. However, we accept this was rather clumsy and could gloss over bias from selective loss to follow-up. We have therefore revised this statement, and also added loss to follow-up under the limitations in the 'Strengths and limitations of this study' box
    Revision: "Selective loss to follow-up *is also* a threat to validity, *with loss to follow-up greatest among participants from the most deprived neighbourhoods. After multiple imputation, findings were still consistent with those from complete-case analysis although this assumes data missing at random given the covariates included in our model.*"

16. General remark: Is the difference in the MSK-HQ-score clinically relevant? Based on previous research the minimal clinically important difference is 5.5 (which is larger than the difference found in this study)? Does this influence the results or the conclusions you can make based on the data in this study?
    Response: The MSK-HQ was originally validated with a within-person minimal clinically important difference (MCID) of 5.5 points. However, there is currently no established threshold for a clinically meaningful between-group difference in trials using the MSK-HQ as a primary outcome. In our recent Orthopathways trial (ISRCTN38924614), a non-inferiority margin of 3.6 points (equivalent to effect size of 0.3, typically classed as small-to-medium) was chosen as a potentially meaningful between-group difference. The

mean between-group difference in MSK-HQ scores between the most and least deprived patients exceeds this but it is largely explained by differences in clinical case-mix. The extent of any widening in inequality gap at follow-up is less than this value for meaningful between-group difference. Therefore we are not seeing a widening that would be equivalent to the effectiveness of care being clearly superior in patients from less deprived neighbourhoods. The slight widening is worthy of note but the main finding is that the inequality gap does not reduce.
Revision: Add to the Discussion: "Are the between-group differences observed in this study clinically important? While a within-person minimal important change of 5.5 points for MSK-HQ has been proposed(35), there is currently no established threshold for a clinically meaningful between-group difference in clinical trials. In a recent trial (ISRCTN38924614), we proposed a value of 3.6 points (which was equivalent to an effect size of 0.3). The mean between-group difference in MSK-HQ scores between the most and least deprived patients in the current study exceeds this but it reduces after accounting for differences in clinical case-mix. Any widening in the inequality gap at follow-up is less than this value and therefore is not statistically significant and below a value that might indicate a clinically meaningful difference in the effectiveness of treatment for those from more deprived neighbourhoods."

17. Also in you study protocol you stated (under primary outcome measures) the aim to determine the percentage of patients who achieve the minimal important change. I can not find anything on this in the manuscript?
Response: Apologies for this omission.
Revision: Number and % of patients meeting MIC value for MSK-HQ at 3 and 6 months added to Table 1

Reviewer: 2

Overall comments and major critiques:

1. Opioid prescription as an indicator of low-value care- this is interesting. I do a great deal of work opioid stewardship and am a chronic pain physician. That said, there is a lot of nuance to the rationale for an opioid prescription, including previous therapies tried, comorbidities, etc. While differential prescriptions by socioeconomic status is interesting, I'm not sure I love terming it low value care.
Response: on reflection we agree – long-term opioid prescription gets closer to an concept of low value care but even this we concede is still a nuanced matter and defining low value care from prescription record data can be challenging. We have amended several sentences, including the statement that was in our limitation section in the Discussion.
Revision: "an indicator of care received" (p2), "We also note that long-term opioid analgesic prescription would have been a *more useful indicator of care quality* than a single opioid analgesic prescription" (p13)

2. Responder bias- while prospective data are challenging a 50% loss to follow up creates meaningful responder bias.
Response: we agree that this level of loss to follow-up must be regarded as a potential source of bias, even when multiple imputation modelling produces similar findings to the complete-case analysis.
Revision: please refer to Reviewer 1 #11 and #15

3. Non-opioid treatments- I appreciate the focus on opioids; however, it would be far more interesting to categorize the other types of treatments given in the first 14 days to better understand what options other patients not receiving opioids are being given and whether this varies by SES. Large categories would be ok---non-opioid analgesics, behavioral therapies, physical therapy, other??
Response: We agree that there could be interest in expanding the treatments to include non-opioid treatments. We did consider these when formulating our pre-specified analysis plan. However, each presents major challenges. In the UK, alternative pharmacological treatments – paracetamol, NSAIDs – are available over the counter. Prescription costs are subsidised in certain groups (e.g. aged over 60 years, receiving income-related welfare benefits). These are likely to introduce a combination of under-estimation and bias which is very difficult to accurately correct. Non-pharmacological treatments are poorly recorded in the structured component of the primary care electronic health record (i.e. given a SNOMED code). Instead the specific nature of the referral may be recorded in free text (which was not available to use for the current analysis) or not recorded at all. Self-referral to NHS physical therapy and use of private providers further complicate the interpretation of data recorded in the primary care EHR. We did include some self-report questions on other treatments but these were subject to missing data as per PROMs and this would therefore require a substantial re-analysis (including re-doing multiple imputation models to include these additional treatment variables and re-estimating all reported parameters from the imputation models).
Revision: we respectfully propose no revision

4. Satisfaction with care while commonly measured is not really a good measure of the quality of care or action patient satisfaction. There are too many variables at play to come out through simple, semi-structured approaches.
Response: we agree that satisfaction with care is a complex and sometimes not very insightful measure. However, it is still frequently used within health services in UK. We think the low proportion of participants reporting dissatisfaction with their consultation provides some food for thought and we had signalled our intention to include a measure of patient experience in our protocol.
Revision: none

5. Abstract- minor point, but would focus on the primary outcomes and provide detail. Secondary outcomes can move to the body of the manuscript.
Response: we have reduced the results on secondary outcomes in the abstract (see also Reviewer 1, #1)

Revision: "Opioid prescription was more common for patients living in the most deprived neighbourhoods ~~(30% cf 19%; fully adjusted odds ratio: 0.69; 95%CI: 0.44, 1.08). Only 6% of patients overall reported being dissatisfied with their consultation~~."

Additional comments:

6. Methods- I see ethnicity but not race. May be differences in the way you handle demographics.
   Response: you are correct – the UK encourages the collection and reporting of self-reported ethnicity rather than race (e.g. UK Government Standards for ethnicity data). The categories we used in this study are those recommended in the UK Government Statistical Services' harmonised standard for ethnicity)
   Revision: no revision
